# Single feature polymorphism (SFP)-based selective sweep identification and association mapping of growth-related metabolic traits in *Arabidopsis thaliana*

**DOI:** 10.1186/1471-2164-11-188

**Published:** 2010-03-20

**Authors:** Liam H Childs, Hanna Witucka-Wall, Torsten Günther, Ronan Sulpice, Maria V Korff, Mark Stitt, Dirk Walther, Karl J Schmid, Thomas Altmann

**Affiliations:** 1 Max-Planck Institute for Molecular Plant Physiology, Am Mühlenberg 1, 14476 Potsdam-Golm, Germany; 2 Potsdam University, Department of Genetics, Institute of Biochemistry and Biology, University of Potsdam, Karl-Liebknecht-Strasse 24-25, 14476 Potsdam-Golm, Germany; 3 Humboldt University, Faculty of Agriculture and Horticulture, Invalidenstr 42, 10115, Berlin, Germany; 4 Leibniz Institute of Plant Genetics and Crop Plant Research (IPK), Corrensstr 3, 06466, Gatersleben, Germany; 5 Max-Planck Institute for Plant Breeding Research, Carl-von-Linné-Weg 10, 50829 Köln, Germany; 6 Swedish University of Agricultural Sciences, Department of Plant Biology and Forest Genetics, Dag Hammarskjölds väg 181, Uppsala, Sweden

## Abstract

**Background:**

Natural accessions of *Arabidopsis thaliana *are characterized by a high level of phenotypic variation that can be used to investigate the extent and mode of selection on the primary metabolic traits. A collection of 54 *A. thaliana *natural accession-derived lines were subjected to deep genotyping through Single Feature Polymorphism (SFP) detection via genomic DNA hybridization to Arabidopsis Tiling 1.0 Arrays for the detection of selective sweeps, and identification of associations between sweep regions and growth-related metabolic traits.

**Results:**

A total of 1,072,557 high-quality SFPs were detected and indications for 3,943 deletions and 1,007 duplications were obtained. A significantly lower than expected SFP frequency was observed in protein-, rRNA-, and tRNA-coding regions and in non-repetitive intergenic regions, while pseudogenes, transposons, and non-coding RNA genes are enriched with SFPs. Gene families involved in plant defence or in signalling were identified as highly polymorphic, while several other families including transcription factors are depleted of SFPs. 198 significant associations between metabolic genes and 9 metabolic and growth-related phenotypic traits were detected with annotation hinting at the nature of the relationship. Five significant selective sweep regions were also detected of which one associated significantly with a metabolic trait.

**Conclusions:**

We generated a high density polymorphism map for 54 *A. thaliana *accessions that highlights the variability of resistance genes across geographic ranges and used it to identify selective sweeps and associations between metabolic genes and metabolic phenotypes. Several associations show a clear biological relationship, while many remain requiring further investigation.

## Background

Many plant species show a wide geographical range across contrasting ecological environments. Reciprocal transplantation experiments of ecotypes from different geographic origins in a common garden, provided evidence for local adaptation within the species range that resulted in fitness differences [1-3]. Local adaptation by means of natural selection affects both the interaction with the abiotic environment, such as soil, temperature or photoperiod, as well as biotic interactions with competitors, pathogens and pollinators [4]. Frequently, plant species show a high level of phenotypic variation among ecotypes in morphology, phenology and biochemistry that is geographically structured [5,6]. The high levels of phenotypic diversity lead to a key question: Which traits are polymorphic due to natural selection and which ones result from the differential fixation of random mutations by genetic drift? Since plants are sessile organisms showing highly structured populations and a propensity for self-fertilization, random drift is expected to play a significant role in local populations. Therefore, one of the central goals of plant evolutionary biology is to disentangle the effects of both processes, to identify traits under selection and the genes controlling these traits.

Natural accessions of *Arabidopsis thaliana *are characterized by a high level of phenotypic variation in morphological, developmental, metabolic, resistance and reproductive traits [6]. Since *A. thaliana *shows a large distribution range throughout the Northern Hemisphere [7] and grows in highly differentiated local habitats, it is ideally suited to investigate the roles of drift and selection in phenotypic variation. For example, flowering time and the vernalisation response show a latitudinal gradient across Europe [8,9], suggesting local adaptation to different day lengths. Early flowering plants with a summer annual habit are predominately observed in Southern Europe whereas late flowering plants with a strong vernalisation response tend to occur in Northern Europe. Major candidate genes controlling flowering time and vernalisation response have been identified and allelic variation segregating at these loci shows evidence of positive Darwinian selection [10-12]. A similar variation in flowering time was also observed in other species, indicating that latitudinal pattern does not result from past historical processes like the re-colonization of glacial habitats and the fixation of new mutations by genetic drift, but resembles local adaptation. Genes that mediate resistance to pathogens (R genes) are also well known to be highly polymorphic in *A. thaliana *[13] and indications of various modes of selection acting on these genes have been obtained [13-20].

In addition to flowering time and pathogen resistance, the utilization of available resources is of central importance for plant fitness. Nutrient availability and the environmental conditions influencing carbon fixation show a high level of spatial and temporal variation. Very substantial variation of biomass accumulation and metabolite composition has recently been observed across *A. thaliana *accessions [21-23] suggesting that local adaptation involves changes in metabolic traits that may result from natural selection. In *A. thaliana*, a positive correlation between above-ground biomass and fecundity was found [24] as well as a strong correlation between biomass and the metabolic profile in a recombinant inbred line population of two genetically divergent accessions [25]. A subsequent analysis of 94 *A. thaliana *accessions uncovered much phenotypic variation in more than 90 metabolites and significant negative correlations between biomass and several metabolites [23]. This work also revealed starch as a major integrator of the metabolic response. Allelic variation at two candidate genes, whose expression is affected by carbon regulation are associated with biomass, thereby providing a target for natural selection. While positive selection has been shown to operate on genes involved in the synthesis of compounds involved in plant defence and to drive diversification in plant secondary metabolism [26], and despite the documentation of significant genetic variation in enzyme activities of primary (and secondary) metabolism in *A. thaliana *[27,28], very little information is available on the extent and mode of selection on primary metabolic traits in plants.

Hybridization-based arrays have been used to address a broad range of questions related to genomic variation [29], such as the identification of natural variation between closely related organisms [30]. Tiling arrays hold sets of microarray hybridization probes that contain both genic and non-genic portions of the genome and can be used to discover polymorphisms throughout the entire genome. Such polymorphisms are called single feature polymorphisms (SFPs) because they differ from a common reference sequence. Although the exact nature of polymorphism in SFPs is difficult to identify they have been established to be a useful alternative to the genotyping of single nucleotide polymorphisms (SNPs) [31].

In the present study we seek to widen the scope of previous genotyping experiments by increasing the number of studied accessions and the density of SFP calling and by broadening the analysis to include representative primary metabolic traits. Two previous studies both investigated ~20 accessions with two different types of arrays: the Affymetrix ATH1 microarray [32] and the Perlegen high density re-sequencing microarray [33]. While the high density re-sequencing arrays provide the best possible resolution available for a hybridization-based approach, high cost limits the number of accessions that can be investigated. The ATH1 microarray on the other hand is limited to ~11 probes per annotated gene. By using the Affymetrix Arabidopsis Tiling 1.0 Array and analysing 54 accessions, we substantially increased the coverage of the *A. thaliana *population analysed by deep genotyping and phenotyping at the cost of not covering29% of the genome and not knowing the exact nature of the underlying polymorphism. We also aim to add to the type of traits studied in *A. thaliana *by including phenotypic and metabolic data. Metabolic traits have been chosen primarily based on their correlation with biomass in a set of 94 accessions [23]. Five of them significantly correlated with biomass (proteins, total amino acids, sucrose, starch and threonic acid) while three metabolites did not show any significant correlation (erythritol, β-alanine, and myo-inositol). Using both types of data, we tested associations between metabolic and growth-related traits such as fresh weight, protein content and metabolite levels and polymorphisms of metabolic genes. In a further step we demonstrate how genome-wide SFPs can be used to detect genomic regions showing signs of recent selection that are associated with phenotypic variation in metabolic traits. Our goal was to identify genomic regions and genes that harbor genetic variation that played a role during the recent history of *A. thaliana *and may have contributed to local adaptation and to use the physiological data to seek indications of the involvement of changes in growth and metabolic features. We achieved this by identification of regions with unusual haplotype structures that may have resulted from the rapid fixation of advantageous mutations and by testing for significant associations with phenotypic variation in the aforementioned growth-related and metabolic traits.

## Results

### Patterns of SFP diversity

Using Affymetrix Arabidopsis Tiling 1.0 Arrays, high density SFP maps for 54 genetically diverse *A. thaliana *accessions were created. An analysis of various possible SFP prediction criteria using a Linear Discriminant Analysis (LDA) reveals that the log base 2 (log_2_)-fold change between Col-0 and non-Col-0 arrays accounts for greater than 99% of the predictive power and is more discriminatory than other tested predictive variables including well established and previously used statistical measures such as Significance Analysis of Microarrays (SAM) or Student's t-test (Additional File 1, Figure S2.7). Based on these results, a threshold to the log_2_-fold change in order to call SFPs based on hybridization signals was applied as it provides the best sensitivity and specificity with the lowest computational time.

**Figure 1:**
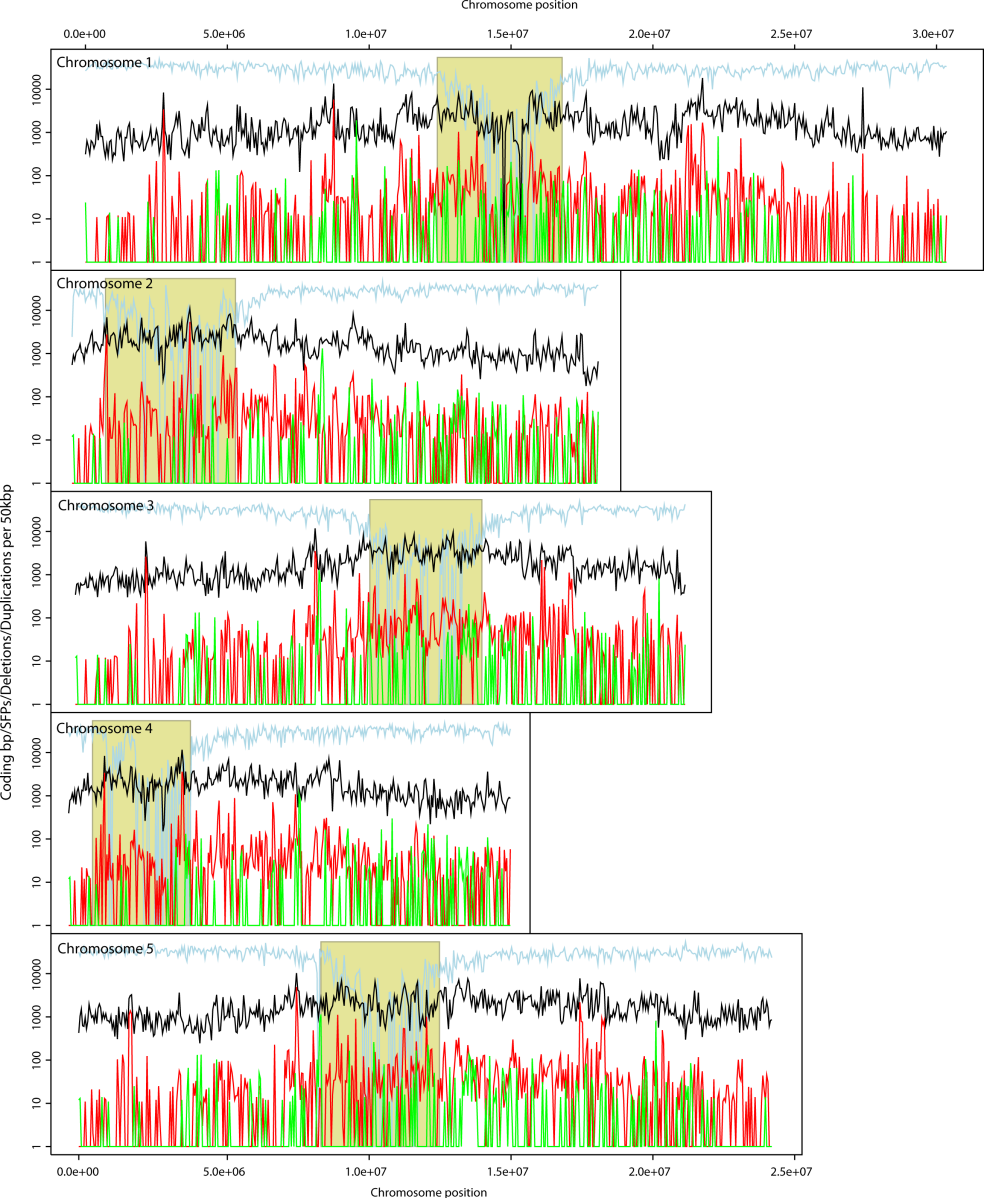
**The density of SFPs and genes across each chromosome of *A. thaliana***. SFP density is calculated for all accessions per base pair (bp) using a sliding window of 50 kbp and the density at each position is plotted on a log scale (shown in black). The gene density, calculated as coding bp per every 50,000 genomic bp is also calculated (shown in light blue). The olive regions show the position of the centromeres. Deletions are shown in red and are defined as 10 probes in a row that were called SFPs (~350 bp). In the figure, the number of deleted bp calculated in a 50 kbp window. We have defined a duplication to be at least 10 probes in a row with a log_2_-fold change of greater than 1 (2-fold increase in signal). In this figure, the number of duplicated bp is calculated in 50 kb windows across the chromosome (green).

The quality of the predicted SFPs for 12 accessions was tested against the 2010 dataset of SNPs determined by sequencing [34] using Receiver Operator Characteristic (ROC) plots. A threshold of -1.5 (2.8-fold decrease in signal) was applied to the log_2_-fold change based on the plots as it produced a suitable False Discovery Rate (FDR - the proportion of false positives among the called SFPs) across the 12 accessions. At this threshold, we were able to predict SFPs at FDRs between 0.45% and 16.0% (Additional File 1, Table S2.1). The False Negative Rates (FNRs - defined as the proportion of false negatives among all polymorphic probes) were estimated to be between 60% and 70% when compared to the 2010 dataset. These relatively high FNRs were due to the stringent threshold set to reduce the number of false positives. False negatives, i.e. probes showing weak differences in signal intensities upon hybridization with perfect match DNA versus mismatch DNA are probably caused by cumulative effects of properties of the probes and the nature of the sequence polymorphisms: The position of an SNP in a probe, the number of SNPs in a probe and the %G+C of probe (Additional File 1, Figures S2.1-2.5) all affected the observed fold changes in signal. The effect of three properties on the probe signal intensity was also tested. By using only probes with a single exact match to the *A. thaliana *genome to call SFPs, we obtained 1,072,557 SFP sites across 54 accessions. The overall SFP density distribution as well as the corresponding gene density across all five Arabidopsis chromosomes is plotted in Figure 1. Based on calculations using the 12 accessions shared with the 2010 dataset, 15% of the predicted SFP sites are estimated to have more than two alleles. It can be expected that this proportion increases with more accessions. On average, 159,313 SFPs were called per accession ranging from 111,259 in Co-3 to 231,383 in H-O-G (Additional File 1, Table S2.2). Our control, Col-0, is used as the reference genome on the array, and produced only 1,560 SFPs, thus confirming the applied SFP calling procedure produces a low FDR.

**Figure 2:**
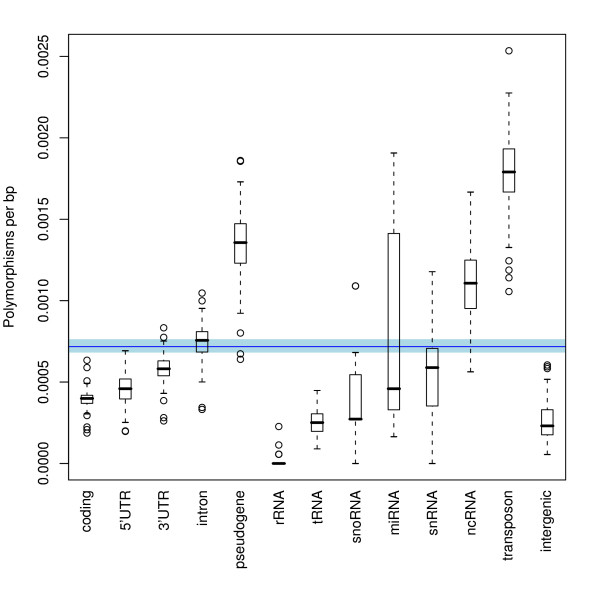
**The presence of SFPs in various genomic structural features is shown as the number of SFPs per base pair for each entity**. The spread of observed values across all accessions is shown in black. The blue band shows the range between the 1st and 3rd quartile while the red line shows the median value. A chi-squared test is used to determine whether the observed amount of SFPs per class was close to the expected number due to random chance. All classes are significantly greater than or less that random (p < 0.05) except for the classes 'intron', 'snRNA' and 'snoRNA'.

Using the TAIR8 genomic annotation information [35], we compared the observed number of SFP sites in genomic features with the expected number under the assumption of a random SFP distribution. For each genomic feature, we calculated the significance of the difference from random expectation using an unpaired Students t-test. As expected, SFP site frequency per nucleotide is significantly reduced in functional regions of the chromosomes (Figure 2). Interestingly, also intergenic regions showed a significantly lower than expected frequency of SFPs. By contrast, there is a significantly higher than expected frequency of SFP sites per nucleotide in non-coding RNA, pseudogenes, and transposons. Introns, miRNA, snRNA and snoRNA show no significant difference from a random distribution.

**Figure 3:**
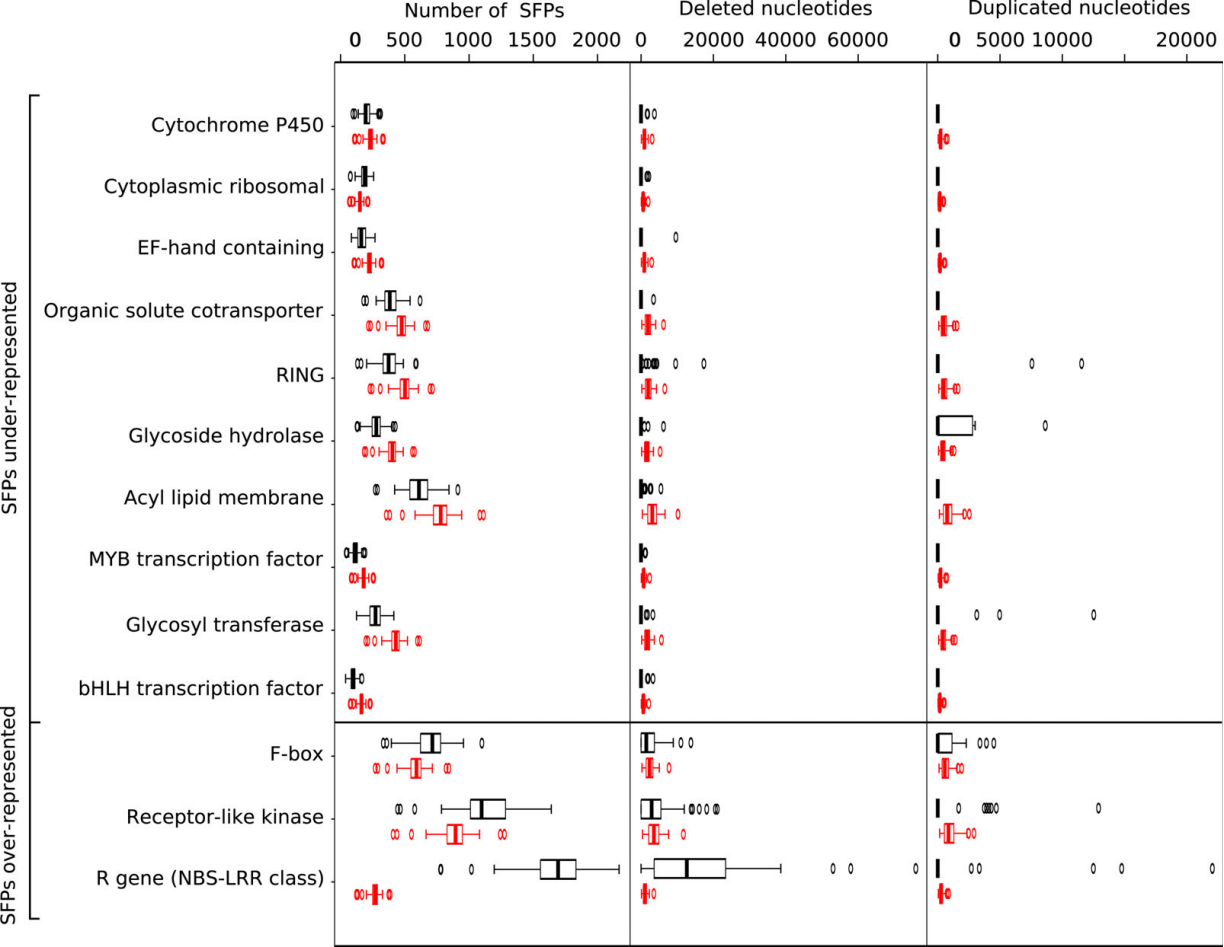
**Number of SFPs in selected gene families**. Of the available gene families in Additional File 1, Table S3.1, 13 are shown here that have been investigated in previous studies for comparison purposes. The observed number (black) of SFPs in a gene family is compared to the expected number (red) due to random chance. Gene families are sorted by the significance of the difference between observed and expected SFP numbers. All gene families show significantly different observed SFPs than expected except for RING genes (Additional File 1, Table S3.1). Deletions follow a similar pattern to SFPs by removing the most nucleotides from resistance, F-box and receptor-like kinase genes but differ by removing fewer nucleotides from cytoplasmic ribosomal genes. Duplications rarely occur in any of the chosen gene families. Although they do occur in resistance genes, the difference between observed and expected is not significant.

For protein coding regions, we collated functional categories from TAIR and NCBI. Of these 40 chosen gene families, 20 contained a significantly greater than random number of SFPs and 18 contained significantly fewer SFPs than expected from a random distribution. Disease resistance, plant defence and signalling genes comprised the majority of the gene families, which were enriched in SFPs. Various transcriptions factors comprised the majority of the top 20 most significant gene families, which were observed depleted in SFPs (Figure 3).

### Deletions and duplications

Using the Affymetrix Arabidopsis Tiling 1.0 Array, consecutive stretches of SFPs were observed that may be caused by an accumulation of many sequence alterations in a certain region resulting in highly deviant non-Col-0 alleles or may be due to deletions. Thus, to classify such regions as deletions it was assumed that strong sequence deviations of non-Col-0 alleles are likely to be confined to short stretches of coding regions and, therefore, consecutive SFP stretches that were longer than the average *A. thaliana *exon were deletions (~350 bp or 10 SFPs). For simplicity, the same threshold was applied to detecting duplications. 3,943 deleted regions with a mean length of 1,086 bp (on average 28 probes in a row) were observed. A small minority of the deleted regions occur across many accessions; however, the majority occur in only one. The accessions show a broad range in the number of deleted regions ranging from 25 in Co-3 to 415 in Dal-12, with a mean of 135 deleted regions per accession (Additional File 1, Table S2.2). Many of the areas with a high SFP density are explained by an abundance of deletions. Of the chosen gene families, the combined length of deletions is largest in Nucleotide Binding Site-Leucine Rich Repeat (NBS-LRR) genes and smallest in bHLH transcription factors (Additional File 1, Table S3.2).

For duplications we chose a threshold of 10 consecutive SFP sites with a fold change of greater than 4-fold as a conservative estimate of duplication. We detected 1,007 duplicated regions, with a mean length of 1,088 bp. Similar to deleted regions, the majority of duplications occurred only in a single accession, while a small minority occurred in many accessions. The accessions also showed a range in the number of duplicated regions from 8 in Blh-1 to 83 in Shakdara, with a mean of 34 duplicated regions per accession (Additional File 1, Table S2.2). Like deletions and SFPs, NBS-LRR and F-box genes had the highest total length of duplicated regions, whereas many other categories were not represented in duplicated regions at all (Additional File 1, Figure S3.3). However, in this case, the enrichment in duplications was not significant for these gene families although it remained significant for MADS-box transcription factors and glycoside hydrolase. It is of note that the same gene classes show a high frequency of insertions and deletions.

### Choice of phenotypic traits

Nine phenotypic traits were selected from a larger data set, including rosette fresh weight (FW) and eight metabolic traits; five of which showed significant correlations with rosette FW [23]. All of the measured traits used in the association study were shown to be normally distributed. Minimum, maximum and median values are summarized in the Additional File 1, Table S1.2.

### Population structure analysis

The analysis of population structure with both the SFP data and independently genotyped SNPs confirms the existence of population structure, even among accessions from Central Europe. It was previously thought that they represent admixture zones since past glaciation events [36,37]. A STRUCTURE analysis [38]http://pritch.bsd.uchicago.edu/structure.html identified four major clusters of accessions (Additional File 1, Figure S1.1-2). These clusters separate the Central Asian, Eastern European, Central/Western European and Iberian accessions. A high proportion of the Central European and Eastern European accessions are admixed individuals, probably reflecting the effect of postglacial population admixture and subsequent genomic recombination in suture zones. The STRUCTURE analysis on SNP data is supported by clustering based on pairwise distance of the SFP data (Additional File 1, Figure S2.8). Distance matrices from both marker systems are highly correlated. The SNP markers also show a significant correlation between geographic and genetic distance although it is weak, possibly because of the large proportion of individuals from Central Europe and the disturbance of the geographic distribution by human agriculture. Taken together, the population structure analysis indicates the need for correction of population structure for this sample in the subsequent association study.

### Association testing

Association testing was performed between 612,249 unique SFP sites, with a minor allele frequency of at least 5%, and all nine phenotypic traits using a general linear model (GLM) as implemented in the TASSEL program [39] providing an empirical distribution of F-scores from which the p-values for each marker were calculated. The effects of population structure were controlled for by using a *Q*-matrix estimated in STRUCTURE and based on 417 SNPs and four populations (*k *= 4) as described in the Additional File 1, Section 1.4. We applied a significance threshold of p = 1e-3 to obtain 12,189 significantly associated SFPs for which we estimate a FDR of 45% based on the empirical distribution as only 5500 random association would be expected at this significance threshold. We chose to tolerate this threshold to provide sufficient numbers of associations for later downstream analysis. However, for later experimental analysis a much more stringent threshold such as p = 1e-4 will yield a more favourable FDR of 20%. For a detailed bioinformatics analysis, we focused on 6,187 metabolic SFP sites, defined as the full set of SFPs detected in the 5,745 metabolic genes annotated in AraCyc, which allows a direct connection to be made between any significantly associated metabolite and metabolic genes associated with that metabolite via known metabolic pathway relationships. After mapping each SFP site to metabolic genes, 15 metabolic genes were found to significantly associate with fresh weight, 12 genes with starch, 10 genes with sucrose, 9 genes with total protein, 40 genes with total amino acids, and 36 genes with threonic acid (Additional File 1, Figure S4.1). For the three metabolites not correlated with fresh weight (β-alanine, erythritol, myo-inositol), 20 metabolic genes were found to associate with β-alanine, 30 genes with erythritol, and 26 genes with myo-inositol. The significantly associated genes, along with the average trait measurement of each phenotype for Col-0 and non-Col-0 can be found in the Additional File 1, Table S4.1. In total, a non-redundant set of 198 metabolic genes associate with the nine traits. Interestingly, 17 genes were associated with more than one trait. In ten cases, the associations with the different traits were by the same SFP, while in the other seven cases the different traits were associated with different SFPs within the same gene. A Mann-Whitney U was used to test the F-scores of metabolic SFPs against the remaining non-metabolic SFPs and showed no significant (p = 0.435) deviation from the empirical distribution implying that metabolic genes in general are not specifically implicated in the phenotypic variation of the tested traits.

To investigate whether genes serving functions other than metabolic ones may be more likely associated with the metabolic traits examined here, we correlated the total association F-score across all metabolic trait associations with the metabolic gene density for all five chromosomes. Generally, there were no significant correlations detectable, except for chromosome 3, for which a positive correlation was found supporting the notion of metabolic genes being more likely associated with metabolic genes than other gene categories on this chromosome (Additional File 1, Figure S4.1).

To provide a broad overview, the pathways significantly over/under-represented in the associations were identified using a Mann-Whitney U test by comparing the F-scores belonging to the SFPs annotated with a particular pathway with those of the remaining metabolic pathways. After multiple testing correction 11 pathways were found to be significantly more associated with their respective traits than the remaining pathways. Four pathways, associating with sucrose, show significant (p ≤ 0.01) deviation from the metabolic SFP F-scores, 4 each for myo-inositol and total protein content, two for starch and threonic acid, and one for erythritol and fresh weight (Additional File 1, Table S4.2). That significantly over-represented pathways were detected may indicate that the FDR for associations is lower than estimated.

### Analysis of selective sweeps

**Figure 4:**
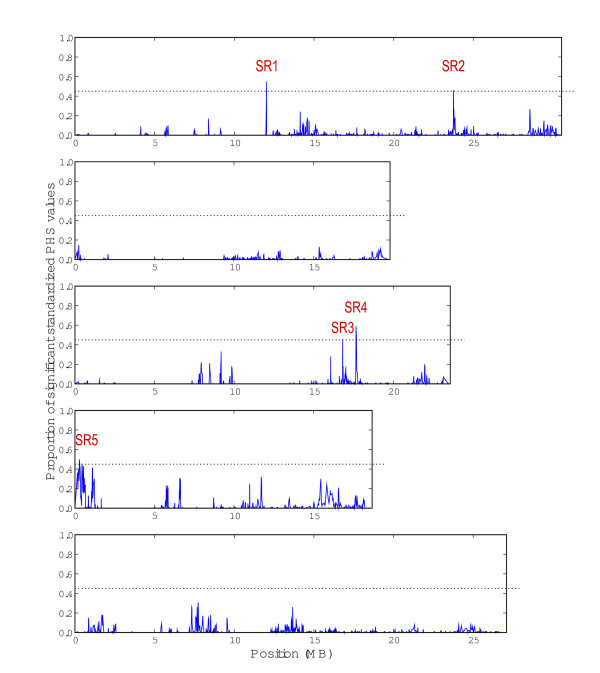
**Map over all sweep candidates**. The graph shows the density of significantly scoring SFPs measured by the proportion of significant SFPs in a sliding window (windows size of 1000 SFPs, offset between windows of 50 SFPs) across the genome. The dashed line represents the cut-off for the definition of sweep candidates. Sweep candidate regions (SR) are denoted in red.

Putative selective sweep regions were identified by detection of unusual patterns of shared haplotypes among accessions based on the notion that genomic regions with selected haplotypes will be longer than regions without selected haplotypes due to hitchhiking of linked variation with the selected mutation [40]. Hence, young and selected alleles will be surrounded by extensive linkage disequilibrium (LD) which produces longer haplotypes, while older alleles would show lower LD due to recombination. Plots of pairwise SFP haplotype sharing indicate regions with high levels of haplotype sharing (Additional File 1, Figure S5.1). We used the pairwise haplotype sharing score (PHS), which was previously applied to *A. thaliana *data [10]. Five significant sweep regions were identified and designated as SR1 to SR5 (Table 1 and Figure 4). These regions show a high proportion of significantly scoring SFPs with unusual haplotype patterns.

**Table 1:** Overview of sweep-candidate regions used for the association analysis

Name	**Chr**.	Focal position	From	To	Length of avg. haplotype sharing
**SR1**	1	12,104,888	12,102,020	12,108,376	6,356
**SR2**	1	23,826,967	23,824,815	23,829,023	4,208
**SR3**	3	16,905,787	16,905,273	16,906,447	1,174
**SR4**	3	17,859,222	17,858,441	17,860,021	1,580
**SR5**	4	198,623	195,197	201,162	5,965

Candidates were called from proportion of high PHS in a sliding window of 2000 SFPs (threshold = 45%). The focal SFP is the highest scoring SFP in the candidate region.

The PHS-test provides the length of the haplotype around each allele and thus the allele with the longer haplotype is likely to be the selected allele. The selected alleles for sweeps regions 1-5 are non-Col-0, Col-0, Col-0, non-Col-0 and non-Col-0, respectively. The neighbouring tendencies of the geographic locations for like-alleles in each of the five detected sweeps were calculated using a Mann-Whitney U test to compare the distances of like alleles in each sweep against those based on 10,000 shuffled variations (Additional File 1, Figure S5.3). This shows that the alleles in sweeps SR2, 3 and 5 have significant neighbouring tendencies with p-values of 3.73E-4, 8.71E-3 and 3.31E-8, respectively. Combined, these results show that SR2 Col-0 alleles cluster in Central Europe in Germany, Switzerland and Czech Republic, the SR3 Col-0 accessions cluster strongly around Western Germany and around the Belarusian/Russian border, and the SR5 non-Col-0 accessions cluster around Western Germany, Austria, Switzerland, and Northern Europe.

The candidate regions range from a length of 145 kb (SR2) to 353 kb (SR4) and the average length of haplotype sharing around the focal SFP ranges from 1.2 kb (SR3) to 6.4 kb (SR1). The genome-wide highest scoring SFP is located in SR3 and, thus, was chosen as focal SFP for this region. For the other SRs, focal SFPs were chosen similarly as the highest scoring SFP in the candidate region. The F-scores of the focal SFPs indicate that only SR5 associates significantly (p = 0.03) with starch. However, the F-scores for all the markers contained within the sweeps, according to a Mann-Whitney U test, show a slight but significant bias (p = 0.0013) towards higher values indicating that markers within sweep regions are more likely to be associated with one of the nine traits than those outside the sweep regions.

## Discussion

Genotyping through hybridization of genomic DNA to genome tiling arrays as applied in this study is characterized by an extraordinarily high degree of multiplexation surpassing all other genotyping methods except the use of whole-genome re-sequencing microarrays [33] and genome re-sequencing using second generation sequencing methods [41]: The Arabidopsis Tiling 1.0 array represents 3,039,991 sites, each 25 nucleotides in length, of the *Arabidopsis thaliana *nuclear genome of which 3,035,275 occur uniquely, and can thus be simultaneously used for identification and localization of sequence variation detected as a substantial deviation of the obtained hybridization signal from that of the reference (DNA of the genotype whose sequence has been used to design the array, Col-0 in the case of *A. thaliana*). Through the use of Affymetrix Arabidopsis Tiling 1.0 arrays, we were able to monitor, in a collection of 54 different non-reference accessions, over one million high quality SFPs. This substantially expands previous data sets [32,33]. Furthermore, we could detect haplotypes and selective sweeps, and associate metabolic and phenotypic traits with the responsible genomic regions. We confirm that SFP monitoring is a viable and cost efficient alternative to SNP typing as proposed by Kim et al. [31] and also demonstrated by Borevitz *et al*. [32], which (in contrast to the latter approach) does not depend on prior knowledge of sequence variation.

Using known sequence information (the '2010 dataset') [34] of 12 *A. thaliana *accessions that were also used in this study, we confirmed that the method used here is suitable for detection of high quality SFPs. The obtained FDRs ranging from 0 and 16% are similar or lower than those reported in previous studies [32,42], which may be due to improvements of the data normalization and/or the metric used for SNP calling or due to higher stringency. The latter is reflected in a relatively high FNR of about 65%. Despite the limitations of the approach, which lie in coverage (71.4% of the genome), obscurity (the exact mutation is not identified and masking of polyallelic variation) and novel/duplicate sequence (unable to analyse novel sequence or the position of duplications), we were able to create high density SFP maps. On average we predicted approximately 160,000 SFPs per accession, which is equivalent to about one SFP per 750 bp of genomic sequence in each pair-wise comparison of an accession with the reference. About 25% to 30% of the SFPs detected in this study were previously identified as sequence polymorphisms in each of the 10 accessions that were also investigated by Clark et al. [43] using resequencing microarrays (Additional File 1, Table S2.3). The limited overlap between the two data sets may be explained partly by the difference in coverage and partly by the different SNP/SFP detection procedures used in the two approaches: The resequencing microarray hybridization data were analysed based on differential hybridization of 8 oligonucleotides for each position in the genome in order to infer base substitutions [43]. This approach is sensitive to further adjacent polymorphisms that interfere with the hybridization to all 8 oligonucleotides [44]. In contrast, SFP calling based on signal intensity comparisons of individual probes between hybridizations of DNA from a particular accession and from the reference is even enhanced at sites with two or more mismatches. The reliability of the data is not only verified by the 2010 dataset but also by a phylogenetic tree that we were also able to construct, based on the SFPs (Additional File 1, Figure S2.8), that groups the accessions according to their geographic location (Additional File 1, Figure S1.1).

The frequency of SFPs among the genomic features shows greater selective pressure on functional regions of the genome especially in coding areas, rRNA and tRNA. SFPs occurring in pseudogenes, transposons and non-coding RNA (ncRNA) appear to be under much reduced selective pressure and, in some accessions, can accumulate to rates as high as one SFP every 400 bp. Of the remaining genomic regions, it is remarkable that non-repetitive intergenic regions also experience a relatively low mutation rate, even lower than that of coding regions. This could be indicative of hitherto largely undiscovered functional regions in the *A. thaliana *genome including regulatory sites.

In addition to individual SFP sites, regions of multiple, immediately neighbouring, polymorphisms were detected. Consecutive stretches of 10 or more polymorphic sites (equivalent to at least 350 bp), all showing reduced signals, were classified as deletions. Across the 54 accessions almost 4000 deleted regions of a mean length of approx. 1.1 kb were detected, most of which occur in only a single accession and on average 135 deletions were detected per accession with a total length of 974 kbp. According to the threshold of 10 consecutive SFP sites, smaller deletions were not considered as they could not be readily distinguished from instances of multiple SNPs or multiple small InDels in close vicinity. Furthermore, as only probes with a unique occurrence in the genome sequence were considered, this analysis was restricted to non-repetitive Col sequence. The fraction of deleted genome sequences detected here can thus be regarded as a conservative minimum number. Conversely, regions were called duplicated if 10 or more adjacent probes displayed greater than a two-fold increase in hybridization signal. Again, the total of about 1000 duplicated regions of an average length of approx. 1.1 kb (across the 54 accessions with a mean of 34 duplicated regions per accession) has to be regarded as a minimum number. The notion of substantial variation in presence or copy number of genomic sequences in *A. thaliana *accessions is supported by the previous observation of 750 InDels larger than 100 bp detected in 263 Mbp shotgun sequence of the Ler-1 accession when compared to the Col-0 sequence, where 93% of the InDels identified in total were shorter than 100 bp [45]. A remarkable variation in nuclear DNA content has been observed through very accurate flow cytometry of 21 *A. thaliana *accessions [46]. This analysis revealed approx. 10% higher DNA content in the accessions with the largest genomes as compared to those with the lowest values. Interestingly, the Col-0 accession, whose full genome sequence has been used to design the Arabidopsis Tiling 1.0 array, had the lowest genome content of the 21 accessions analysed. Throughout the present study we observed fewer duplications than deletions in the 54 accession analysed. While it remains to be analysed how much of the variation in genomic DNA across accessions is accounted for by repetitive DNA, our results indicate that the additional DNA in the accessions with larger genomes is not merely caused by increased copy numbers of sequences present in the Col-0 accession (which should have been detected as a much higher incidence of duplications). Thus, a substantial fraction of the genome complement of the *A. thaliana *species represented by the additional nuclear DNA in many accessions is apparently yet uncharacterized.

While the detected duplications are so rare that they do not affect any of the analysed gene families of in most accessions, their occurrence in some functional categories is particularly striking in a few accessions: For example, 22,000 bp of the NBS-LRR disease resistance genes are duplicated in accession El-0. This is significantly more than the expected 307 bp. In a similar case, 37,000 bp of the MADS-box transcription factors are duplicated in the Wei-1 accession.

The obtained polymorphism information was used to investigate the degrees of diversity among a broad range of functional gene classes. Whether they are duplications, deletions or individual SFPs, there are a much greater number of polymorphisms than expected at random in disease resistance and defence related gene families (Additional File 1, Table S3.1-3). Across a broad geographic range, an encounter with a large spectrum of pathogens can be expected and the high occurrence of polymorphisms in the resistance genes reflects the need for defence genes to adapt to the local pathogens and environmentally specific challenges. Among the genes that have the most significantly greater number of SFPs than expected, are the Nucleotide Binding Site-Leucine Rich Repeat (NBS-LRR), anthranilate synthase, F-box, terpene and monooxygenase gene families. All these gene families are either directly involved or implicated in plant defence. Transmembrane receptor, thioredoxin, leucine-rich repeat kinase, receptor-like kinase, and wall-associated protein kinase gene families and a subset of the proteins encoded by the other genes families identified to be highly polymorphic are involved in inter- and intracellular signalling potentially reflecting variation in responses to external and internal signals that may be relevant for local adaptation. F-box, NBS-LRR and transmembrane receptor genes have been previously identified as having a high number of polymorphisms [32,43]. On the other hand, many transcription factors, calmodulin binding genes, helicases, ABC transporters, glycosyl transferases, and acyl lipid metabolism genes contain significantly fewer SFPs than expected. Proteins encoded by these genes thus appear to be involved in more conserved processes.

As a first step towards investigating the viability of genome-wide associations testing for the identification of functionally relevant sequence variation, we subjected all SFP sites to association tests with nine phenotypic traits including total biomass (fresh weight), starch content, total protein content, total amino acid content, and contents of five individual metabolites (sucrose, threonic acid, erythritol, myo-inositol, and β-alanine, with particular focus on those which were located in genes annotated with metabolic functions according to AraCyc. In total 198 significant gene-trait associations were detected. In some cases the non-Col-0 allele may actually be a heterogeneous group and elevated numbers of false negatives are thus expected. This would be the case when the sub-groups show both raised and lowered trait measurements relative to Col-0 thus obscuring the signal even though they may be significant when associated individually. Furthermore, associations caused by population structure alone are difficult to identify, here we used the method by Yu [47] as implemented in TASSEL. However, there may still be residual population structure confounding the data.

Several of the gene functions are directly biologically related to the trait with which the gene is associated. These include various amino acid synthesis and degradation genes associated with total amino acids, β-alanine biosynthesis and putrescine biosynthesis with β-alanine (the degradation of putrescine products produces β-alanine) and starch degradation with starch. Although these annotations may accurately describe the association, it is probable that it is the combined effect of many genes that produce variation in the associated phenotype. Nevertheless, these examples indicate that SFP-based association testing leads to the identification of polymorphisms in genes with (proposed) metabolic function, that are worth to be further investigated as they may lead to the discovery of functionally relevant variation.

Based on the minimum paths calculated between the metabolic genes and the significantly associated metabolites, a considerable number of gene-metabolite pairs were found to have no known connection. For example the associations between *AT1G16780 *(UDP-glucose biosynthesis) and erythritol, *AT2G43760 *(molybdenum cofactor biosynthesis) and myo-inositol, or *AT1G47260 *(aerobic respiration) and threonic acid. While some of these associations may be false positives, at least a subset of them may hint to hitherto unknown or un-annotated connections between the gene products and the associated metabolites. An observed association may however also be caused by linkage disequilibrium and thus a neighbouring gene or mutation may be responsible for the change in metabolite level. A further interesting possible process causing seemingly functionally unrelated gene-metabolite associations may be co-evolution. In previous studies a certain combination of a large number of metabolite levels rather that individual metabolite levels were found to correlate well with plant biomass [25] and multiple metabolite - metabolite correlations were observed [21,23]. Thus, adaptation to certain environments may involve numerous metabolic modifications that may lead to detectable associations although the gene products and the associated metabolite reside in unlinked metabolic pathways and thus are only connected through higher level functional integration. Such considerations may warrant follow-up studies on the metabolites of the pathways the detected gene products are involved with and on their correlations with the associated compounds identified here.

Using the pairwise haplotype sharing score (PHS) as criterion to identify regions with signs of recent selection, five candidate selective sweep regions, SR1 through SR5 were identified. The FNR leads to an overestimation of Col haplotype length and an underestimation von non-Col haplotypes; however, three of our sweeps are non-Col alleles. Due to the interference of the FNR on sweep detection, the sweep analysis should be considered preliminary. The applied PHS test was not able to show significant signatures of selection in the candidate regions previously suggested by Clark et al. [33]. Although we found extensive haplotype sharing in one of their candidates on chromosome 1, this region showed no clear peak in PHS density even if we relaxed the threshold for significant scores. This discrepancy might be caused by the different composition of our sample, but the most important difference may be the method used for the identification of sweep candidates. Clark et al. simply identified regions with a low diversity over a long range. The PHS test compares the length of the different haplotypes around a core allele and, additionally, accounts for the population structure and local recombination rate. Hence, the PHS test represents a more rigid measurement to identify selective sweeps then the approach by Clark et al. [33]. The power of the PHS test to detect true positive signals of selective sweeps is demonstrated by the identification of candidate region SR5. This region includes the gene *FRIGIDA*, which is a major regulator of flowering time and which was previously identified to evolve under selection [2,10].

A relative overrepresentation of SFPs with extreme PHS values (multiple testing adjusted p = 1.6E-10) was observed in NBS-LRR protein-encoding genes. This suggests that these genes are preferred targets of selection in *A. thaliana *in addition to being highly polymorphic (see above). The other functional class significantly enriched in extreme PHS values (p = 0.04) is the receptor-like kinases genes-category. Interestingly, although cytoplasmic ribosomal protein genes and F-Box genes are among the most highly polymorphic genes (Additional File 1, Figure S5.4-5), none of the chosen gene families show significant enrichment or depletion in the sweep. Due to the obscurity of the SFPs, it is hard to exclude that the identified sweep regions may in fact represent multiple independent sweeps in the same region. However, it has been shown that the power to detect sweeps based on their haplotype structure is reduced if more than one sweep occurs in the same region [48]. Thus, the identified candidates most likely represent single sweeps and can be regarded as biallelic for the associations although the strength may be overestimated.

The 54 non-Col accessions investigated here have been thoroughly characterized with respect to their biomass accumulated during growth in controlled condition and they have been investigated for more than 90 metabolic parameters including protein and chlorophyll contents, starch, a large number of low molecular weight metabolites and several enzyme activities [23]. Substantial variation in biomass accumulation and many of the metabolic traits as well as complex relations between them were observed raising the question whether or not this is a result of recent selection related to adaptation that occurred during the recent evolution of *A. thaliana*. As a first step towards addressing this question, associations were tested between the selected haplotypes of the sweeps and plant fresh weight as well as eight metabolic traits chosen as representatives of different groups of cellular components including structural and storage compounds as well as central metabolic intermediates. A significant association was detected between one of the five sweep regions (SR5) and starch. Furthermore, SR5 coincides with a strong biomass QTL and a metabolite QTL hot spot detected in the Col-0/C24 RIL and IL populations [49] and this region furthermore confers heterosis of biomass and 23 metabolites [49,50]. Although SR5 exhibits a considerable population structure, we do not assume that the association is confounded by it. The signal is retained after different corrections for population structure in the PHS test and the GLM and is further supported by the presence of the QTL. This indicates that at least some of the metabolic differences between the accessions analysed here occurred as a result of or are coincidental with recent selection. While further analysis will be required to investigate which traits controlled by which genes within the sweep regions were driving the selection, the observations made in this study provide a hint that genes within the sweep regions may contribute directly or indirectly to the metabolic diversity represented in the *A. thaliana *population. These regions do not include known metabolic genes and the results may thus open the opportunity to identify genes with hitherto unknown enzymatic or regulatory function.

As sweeps are likely due to positive selection and thus should have an observable impact on phenotypic variation, it is expected that a significantly greater number of sweep SFP sites should associate with the measured traits than those that are randomly chosen and the sweep marker-trait association result presented supports this hypothesis. However, only a small number of phenotypes were tested. To truly test this hypothesis, a larger number of phenotypes need to be included in the association mapping.

Due to the large proportion of false negatives, the size and number of the predicted sweeps are likely to be reduced along with the number of associations. The false negatives are not likely to impact the quality of the associations and sweeps as they reduce existing signal rather than add noise. That is, in association mapping the correlation between high/low metabolite levels and presence/absence of a SFP can only be reduced by false negatives and similarly for detecting sweeps. False positives can increase the noise and lead to false associations and sweeps. However, these are estimated to be called at low rates and as they are randomly distributed throughout the genomes, their impact on the quality of the results is further reduced.

### Natural selection mapping versus molecular genetics

We have identified genomic regions with genetic variation that are associated significantly with growth-related metabolic traits in greenhouse conditions. Even though the phenotypic analysis was not conducted under natural conditions or in nature itself, the associations strongly suggest the location of variants that have a robust effect on complex traits. It can not be excluded, though, that in nature or evolutionary history, a different phenotypic effect of the selected region may have been selected. Furthermore, the region is large, and the phenotypic effect could hitchhike with the selected mutation, that in fact is responsible for another type of phenotypic variation.

A critical issue in natural selection mapping is the age of the sweeps. Even though phenotypic variation is associated with the sweep, it may not necessarily be the trait that evolved under selection, but may even be a (slightly) disadvantageous trait. If the sweep occurred recently, there may not have been much time for compensatory mutations to neutralize this phenotypic effect [51].

Nevertheless, this analysis represents a precedence of a viable procedure to identify genomic regions carrying functionally relevant variation in (probably regulatory) genes, which is potentially involved in adaptation. On the one hand these results open the exciting opportunity to identify and characterize the genes responsible for the phenotypic variation of growth-related metabolic traits through the investigation of the sequence variation of the identified regions across the accession population and the functional analysis of the encoded genes and their alleles through phenotypic characterization of genetic substitution lines, mutants, and transgenic plants grown in a variety of conditions. They also indicate how powerful this approach will become in the near future upon availability of deep genotype/re-sequencing information and phenotypic data of hundreds of *A. thaliana *accessions.

## Conclusions

By hybridizing genomic DNA extracted from 54 *Arabidopsis thaliana accessions *to Arabidopsis tiling microarrays, we identified a large set of Single Feature Polymorphisms, thereby generating a high density polymorphism map of *Arabidopsis thaliana. *Its analysis revealed a pronounced variability of resistance genes across geographic ranges and identified selective sweeps and associations between metabolic genes and metabolic phenotypes. Several associations show a clear biological relationship, while many remain requiring further investigation.

## Methods

### Creating the dataset

The materials and methods used for accession selection, plant material preparation, phenotypic analysis, DNA preparation and array hybridization are presented in the Additional File 1, Sections 1.1-3. Measurements of linkage disequilibrium and population structure are presented in the Additional File 1, Section 1.4. Tiling array analysis and SFP calling are presented in the Additional File 1, Section 1.5.

### SFP annotation and analysis

Statistically significant enrichments or depletions in annotated genomic regions (coding regions, intergenic regions etc...) were detected by comparing the observed number of SFPs in a region to the expected number assuming that SFPs are distributed proportionally to the total size of the region. Gene families and functional annotations were obtained from TAIR and NCBI. Forty gene families were chosen based on families analysed in previous studies [33] and the gene families available in TAIR. Statistical significance was evaluated using an unpaired Students t-test.

### SFP-trait associations

To reduce the multiple testing problem, only SFPs that lay in metabolic genes (as annotated by AraCyc 11/03/2009) were used in association testing. Marker-trait associations were calculated using general linear models (GLM) [47] as implemented in the Tassel program [39]. The GLM requires a population matrix (*Q*), which was obtained from the program Structure 2.2.

The GLM (general linear model) function of the Tassel program was applied with the Structure results for *k *= 4 to determine the significance of marker-trait associations for following traits: fresh weight, protein, amino acids, sucrose, starch, myo-inositol, *β*-alanine, threonic acid and erythritol. Associations were tested between all SFP sites and phenotypic traits simultaneously providing an empirical distribution from which p-values were calculated[52]. Phenotype data were calculated as least squares means (LSM) values over the experiments. The GLM that was used for the calculations was (Eq. 1):(1)

where *v *is the dependent variable, *M *is a marker effect, *Env *is an effect of environment of different experiments, *Q *is population structure cofactor, *E *is experimental error. A least squared means (lsmeans) test that shows the change in phenotypic measurement between Col-0 and non-Col-0 genotypes is included in the Tassel analysis.

### Association annotation

Each SFP was annotated with the metabolic pathways of the gene they lay within according to AraCyc [35]. To reduce the effect of potential biases introduced by linkage disequilibrium, all analyses were conducted using whole genes rather than the SFP themselves.

The minimum paths between the gene products and the associated metabolites were determined using the AraCyc reactions database [35]. When calculating the minimum path, common and readily available molecules such as water, carbon dioxide, oxygen, free protons, ATP, NADH etc. were ignored.

Each trait associated significantly with a number of different genes, many of which belonged to the same pathway. Pathways of interest were tested using a Mann-Whitney U test to show if they were over-represented for each trait. The number of tested pathways was limited to 13 of particular interest, out of a possible 317, to reduce the effect of multiple testing which was corrected for using Benjamini-Hochberg correction [53].

### Identification of selective sweeps

The detection of selective sweeps followed the approach of Toomajian et al. [10]. The PHS statistic [10] was computed for all SFPs with a minor allele frequency of at least 5%. This test for selection corrects for the population structure by taking the pairwise similarity of accessions into account, therefore it should be suitable for our structured sample from natural populations. Additionally, it accounts for the local recombination rate by using genetic distances between the markers. For this conversion, we fitted a polynomial curve to 253 markers for which physical and genetic position are known, as previously done [54]. Since SFPs in close proximity tend to be in high LD, the PHS test assigns similar scores to these markers. To avoid the redundancy of such nearby markers and the over-counting of high SFP density regions only the highest scoring SFP among highly linked neighbouring SFP pairs (r^2 ^> 0.5 and r positive) was included in the following analysis (standardisation, identification of sweep candidates) [10]. The scores were then standardized for their allele frequency *f *like follows (Eq. 2):(2)

where *SD*_*f *_and *median*_*f *_refer to standard deviation and median, respectively, computed for all alleles with a frequency of *f*; the median is used instead of the mean because it is less sensitive to extreme values.

To identify sweep candidate regions, the density of top 0.5% standardized scores were determined by calculating the proportion of such outliers in a sliding window along the genome (window size of 1000 SFPs and offset of 50 SFPs between adjacent windows). Windows with an outlier proportion of more than 45% were denoted as sweep candidates and within windows the highest scoring SFP was taken for the associations. The identified sweeps are represented as a focal SFP site that best resembles all the SFP sites in the sweep.

#### Sweep associations

Once identified, only the focal SFP sites in each sweep were used to test for association between the sweeps and the phenotypic traits thereby reducing the chance for spurious associations based on linked SFPs.

## Availability

All data presented in this study are available from http://sfp.mpimp-golm.mpg.de.

## Abbreviations

SFP: Single Feature Polymorphism; SNP: Single Nucleotide Polymorphism; FDR: False Discovery Rate; FNR: False Negative Rate; NBS-LRR: Nucleotide Binding Site-Leucine Rich Repeat; PHS: Pairwise Haplotype Sharing Score.

## Authors' contributions

LC developed and implemented the SFP calling methods, performed all computational analyses, interpreted the results and wrote the manuscript, HWW and MKS performed all experimental work and conducted the association study, TG and KS performed the sweep region identification and wrote the manuscript, RS and MS generated and supplied the phenotypic data, DW analysed and interpreted the results and wrote the manuscript, TA initiated and co-ordinated the study, interpreted the results and wrote the manuscript. All authors have read and approved the final version of the manuscript.

## Supplementary Material

Additional file 1 **Supplementary Material, Methods and Additional Analyses**. This file contains the methods and materials for plant growth, metabolic measurements and SFP calling. Also included are accession specific details (number of called SFPs per accession etc...), annotation for both the polymorphisms and associations and sweep annotation. Click here for file
