# Supplementary material for: Single feature polymorphism (SFP)-based selective sweep identification and association mapping of growth-related metabolic traits in Arabidopsis thaliana
Source: BMC Genomics. 2010 Mar 20;11:188. doi: 10.1186/1471-2164-11-188 (PMC2850358; doi:10.1186/1471-2164-11-188)
Supplement: Additional file 1 — Supplementary Material, Methods and Additional Analyses. This file contains the methods and materials for plant growth, metabolic measurements and SFP calling. Also included are accession specific details (number of called SFPs per accession etc...), annotation for both the polymorphisms and associations and sweep annotation. [file 1471-2164-11-188-S1.DOC]

# Experimental preparation

## Selection of accessions

For this study, a total of 54 accessions representing native ranges in Central Europe, Southern Europe, Asia and Eastern Europe were chosen from a larger set that had been subjected to phenotypic characterisation (Sulpice et al., submitted). The *A. thaliana* accessions used in this study were obtained from various sources: Col-0 from G. Rédei (Univ. of Missouri-Columbia, USA); Ler-1 from M. Koornneef (Wageningen University, Netherlands); Te-0 from S. Misera (Institut für Pflanzengenetik und Kulturpflanzenforschung, Gatersleben, Germany); Bor-4, Est-1, Lov-5, NFA-8, from D. Weigel (Max Planck Institute Tübingen), Ak-1, Bur-0, Enkheim-D, Jea, Mh-1, Oy-0, Petergof, Pyl-1, Shakdara, Stw-0, and Ta-0 from the Versailles stock centre. All others were obtained from the Nottingham Stock Centre (NASC), through which all accessions are now available. Accessions were homogenised by single-seed propagation and were bulk-amplified prior to the analyses conducted [1]. Table S1.1 lists all the chosen accessions and their geographic locations.

## Plant material preparation for phenotypic analysis

### Plant growth conditions

Accessions were grown in short-day conditions (8/16h light/dark) in moderate light and well-fertilised soil to apply a moderate carbon deprivation, and harvested during the last hour of the day, 5 weeks after germination when they were still in the vegetative growth phase. Rosette fresh weight at the harvest time was measured as an indicator of biomass. To assess potential links between biomass and primary metabolism, six metabolic traits significantly correlating with biomass and two that that did not were selected (Sulpice et. al. submitted). Protein, total amino acids, β-alanine, sucrose, starch, threonic acid, erythritol and myo-inositol were measured as representatives of structural and storage components (see below). Phenotype data were calculated as least squared means values over the experiments. Phenotyping for the traits used in the association mapping was carried in 6 to 8 independent experiments, in particular for fresh weight, protein, amino acids, sucrose and starch in 8 experiments and for myo-inositol, β-alanine, threonic acid and erythritol in 6 experiments.

### Metabolic trait analyses

Metabolic traits were analysed as described by Sulpice et al. (submitted). In brief: Chemicals were purchased as described by Gibon et al. [2] and total protein, starch, sucrose, total amino acids were assayed as described by Cross et al. [3]. β-alanine, threonic acid, erythritol and myo-inositol were determined by gas chromatography (GC-MS) coupled to mass spectrometry. Metabolite extraction for GC-MS was carried out on the exact same samples as used for enzymes and metabolites determined by spectrophotometric methods as described previously [4]. 50 mg of *A. thaliana* shoots were homogenised using a ball mill pre-cooled with liquid nitrogen. Derivatisation and GC-MS analysis were carried out as described previously [5]. The GC-MS system was comprised of a CTC CombiPAL autosampler, an Agilent 6890N gas chromatograph and a LECO Pegasus III TOF-MS running in EI+ mode. Metabolites were identified in comparison to database entries of authentic standards [6].

| Accession | Country | Region | Latitude | Longitude | Accession | Country | Region | Latitude | Longitude |
| --- | --- | --- | --- | --- | --- | --- | --- | --- | --- |
| Ak-1 | Germany | C. Europe | 48.00 | 8.00 | **Ler-1** | Poland | E. Europe | 51.31 | 12.10 |
| Bay-0 | Germany | C. Europe | 49.40 | 11.00 | **Lov-5** | Sweden | N. Europe | 58.65 | 16.42 |
| Bch-1 | Germany | C. Europe | 53.50 | 10.50 | **Mh-1** | Poland | E. Europe | 53.31 | 20.12 |
| Bla-11 | Spain | S. Europe | 41.42 | 2.14 | **N13** | Russia | Asia | 61.60 | 34.25 |
| Blh-1 | Czech Rep. | E. Europe | 48.83 | 16.74 | **NFA-8** | UK | W. Europe | 51.25 | 0.41 |
| Bor-4 | Czech Rep. | E. Europe | 49.40 | 16.22 | **Nd-0** | Germany | C. Europe | 47.40 | 8.20 |
| Bsch-2 | Germany | C. Europe | 50.00 | 8.50 | **No-0** | Germany | C. Europe | 51.10 | 13.30 |
| Bu-2 | Germany | C. Europe | 50.50 | 9.50 | **Nw-3** | Germany | C. Europe | 50.30 | 8.40 |
| Bur-0 | Ireland | W. Europe | 53.07 | -9.04 | **Old-1** | Germany | C. Europe | 53.00 | 8.00 |
| Cl-0 | Unknown | Unknown | NA | NA | **Ove-0** | Germany | C. Europe | 53.40 | 8.40 |
| Co-3 | Portugal | S. Europe | 40.50 | -8.50 | **Oy-0** | Norway | N. Europe | 60.23 | 6.13 |
| Col-0 | Poland | E. Europe | 52.44 | 15.15 | **Petergof** | Russia | Asia | 59.88 | 29.90 |
| Da-0 | Germany | C. Europe | 50.00 | 8.50 | **Pr-0** | Germany | C. Europe | 50.10 | 8.70 |
| Da(1)-12 | Czech Rep. | E. Europe | 49.80 | 15.50 | **Pt-0** | Germany | C. Europe | 53.50 | 10.50 |
| Dijon-M | Russia | Asia | 55.75 | 37.62 | **Pyl-1** | France | W. Europe | 44.39 | -1.10 |
| Ei-2 | Germany | C. Europe | 50.30 | 6.30 | **Rak-2** | Czech Rep. | E. Europe | 49.00 | 16.30 |
| El-0 | Germany | C. Europe | 51.30 | 10.00 | **Sap-0** | Czech Rep. | E. Europe | 49.80 | 14.40 |
| Enkheim-D | Ukraine | Asia | 48.00 | 37.80 | **Shakdara** | Tajikistan | Asia | 37.41 | 71.64 |
| Er-0 | Germany | C. Europe | 49.50 | 11.00 | **St-0** | Sweden | N. Europe | 59.19 | 18.03 |
| Est-1 | Estland | E. Europe | 58.50 | 25.50 | **Stw-0** | Russia | Asia | 52.57 | 36.04 |
| Goe-2 | Germany | C. Europe | 51.53 | 9.93 | **Ta-0** | Czech Rep. | E. Europe | 49.40 | 14.70 |
| Got-7 | Germany | C. Europe | 51.53 | 9.93 | **Te-0** | Finland | N. Europe | 60.30 | 25.50 |
| H-O-G | Tajikistan | Asia | 39.00 | 71.00 | **Ts-1** | Spain | S. Europe | 41.74 | 2.74 |
| Hl-3 | Germany | C. Europe | 51.50 | 9.50 | **Wei-1** | Switzerland | C. Europe | 47.40 | 8.40 |
| Je54 | Czech Rep. | E. Europe | 49.80 | 15.50 | **Wil-1** | Lithuania | E. Europe | 55.00 | 25.00 |
| Jea | France | W. Europe | 43.60 | 7.04 | **Ws-3** | Belarus | Asia | 52.50 | 30.00 |
| Kae-0 | Austria | C. Europe | 46.50 | 14.50 | **Zue-1** | Switzerland | C. Europe | 47.36 | 8.55 |
| Kondara | Tajikistan | Asia | 38.55 | 68.80 |  |  |  |  |  |

**Table S1.1:** Geographic locations of the 54 accessions. Accessions were chosen to represent native ranges in Central Europe, Southern Europe, Eastern Europe and Asia. There were, in total, 22 accessions from Central Europe, 12 from East Europe, 9 from Asia, 4 from North Europe, 3 from South Europe, and 1 unknown.

| **Trait** | **Min** | **Max** | **Mean** |
| --- | --- | --- | --- |
| Total amino acids | 13.74 | 31.75 | 19.1 |
| β -alanine | 0.29 | 1 | 0.49 |
| Erythritol | 0.25 | 1.58 | 0.71 |
| Rosette fresh weight | 128.34 | 460.42 | 247.15 |
| Myo-inositol | 1.14 | 3.97 | 2.09 |
| Total proteins | 15.19 | 23.9 | 18.46 |
| Starch | 26.95 | 54.78 | 38.9 |
| Sucrose | 3.28 | 5.23 | 4.02 |
| Threonic acid | 1.4 | 3.87 | 2.43 |

**Table S1.2:** Numeric range of measured phenotypic traits. Measurements for each trait are normally distributed. Six of the traits (total protein, starch, threonic acid, total amino acids, β-alanine and sucrose) are significantly correlated with fresh weight while two (erythritol and myo-inositol) do not.

## DNA preparation for tiling array hybridisation

### Plant growth conditions

Growth conditions for phenotypic analyses were exactly as previously described by Cross et. al. [3]. Briefly: Seeds were germinated and grown for the first 7 days with a day length of 16 h, temperature of 6°C at night and 20°C during daytime, humidity 75%, and luminosity 145 µmol m^-2^ s^-1^. After 7 days, seedlings were transferred to a phytotron. Growth was continued in an 8-h-light/16-h-dark regime at temperatures and humidities of 16°C and 75% at night and of 20°C and 60% during the day. Illumination was 145 µmol m^-2^ s^-1^. At the age of 2 weeks, plants of average sizes were transferred to pots of 6 cm in diameter (5 plants per pot). Plants were switched to a controlled small growth chamber after 1 further week in the short-day conditions outlined above for 2 weeks more. Day length was then 8 h, temperature a constant 20°C, and illumination an average 125 µmol m^-2^ s^-1^. Plants were watered daily. Harvests of 15 plant rosettes were performed at the end of the light period. Each sample typically contained three rosettes, equivalent to 500 mg fresh weight, depending on the accessions. The entire sample was powdered under liquid nitrogen and stored at -80°C until its use.

### Extraction of genomic DNA

25 ml of preheated CTAB buffer were added to approximately 4g of plant tissue, ground in a Retch-mill in liquid nitrogen and incubated for 20 min at 65˚C. 10 ml of chloroform:isoamyl alcohol (24:1) were added to the samples, which were then incubated for 20 min at room temperature followed by centrifugation for 5min at 3000 rpm. The upper phase was transferred to a new Falcon tube and 17ml of isopropanol were added, followed by incubation for 10min on ice and centrifugation for 5min at 3000 rpm. The upper phase was discarded and 4ml of H2O were added to the pellet and mixed. 4ml of 4M LiCH3COO were added followed by an incubation step for 20min on ice and then cleared by centrifugation for 10 min at 3000 rpm. The upper phase was transferred to a new Falcon tube and 16ml of 96% EtOH were added and incubated at 4˚C for 1 hour. The sample was pelleted by centrifugation for 20min at 4000rpm and dried. Afterwards the pellet was re-suspended in 900µl H2O, transferred to two new Eppendorf tubes (2ml) and 140µl of RNAse (10mg/ml) were added to the each sample and incubated for 30min at 56˚C. 130µl of 5M NaCl were added to the samples followed by centrifugation for 5min at 12000 rpm. The upper phase was again transferred to a new Eppendorf tube and 360µl of phenol/chloroform were added gently mixed and spun-down by centrifugation step for 5min at 14000 rpm. In the next step, the upper phase was transferred to a new tube mixed with 1ml of chloroform:isoamylalkohol (24:1) and cleared by centrifugation step for 5min at 14000 rpm. The whole step was repeated and the upper phase was transferred to a new Eppendorf tube and mixed with double volume of 96% EtOH following by incubation for 5 min on ice. The mix was centrifuged for 5min at 14000 rpm and the upper phase was discarded. The pellet was washed with 70% EtOH, shortly centrifuged and the upper phase was discarded. The last step was repeated. Samples were placed on a thermo block at 37˚C for drying. The pellet was dissolved in 50µl of H2O. The DNA quality was tested using a NanoDrop photometer (Thermo Scientific). Only samples with 230/280 factor > 1.85 were used for further analysis.

### DNA digestion and labelling

3.5µl 10x One-Phor-All Buffer (GE Healthcare), 2.1µl 25 mµ CoCl and 1µl 1:3 1U/µl DNase (Promega) were quickly added to 25 µg genomic DNA and was filled up to a total volume of 35µl with H2O. Digestion was carried out for 4 min at 37˚C. To stop the reaction, samples were incubated for 15 min at 95˚C and then cooled down to 4˚C.

The quality of digested products was checked by 2% agarose TBE gel electrophoresis. Samples were classified as well digested if the smear of DNA fragments was about 50 base pairs. 2µl of 20 U/µl Terminal deoxynucleotidyl transferase and 2µl of Biotin-N6-ddATP (Enzo Life Sciences Inc.) were added to 20 µg of digested DNA and incubated for 1 h at 37˚C, followed by a cooling step to 4˚C. The reaction was carried out in a dark room.

### Array hybridisation

Labelled DNA samples were sent to the RZPD (Berlin) service unit for hybridisation. Affymetrix array hybridisation was carried out according to the manufacturer’s standard instructions for expression array hybridisations. For each accession, a total of three independent DNA samples were used. Three reverse arrays were hybridised (one for each of the biological replicates) and a fourth forward array was hybridised using a DNA mixture of the three biological replicates.

## Linkage disequilibrium and population structure

The accessions were genotyped with 460 SNP markers: 149 framework SNPs assembled in the frame of the A. thaliana “HapMap” project, (J. Borevitz, pers. communication; see http://naturalvariation.org/hapmap and links given therein) and 311 SNPs with intermediate allele frequency selected by N. Warthmann et al. [7]. SNP genotyping was carried out at Sequenom Inc (San Diego, CA, USA). Microsatellite typing was perform using Li-Cor 4300 (LI-COR Biosciences GmbH, Germany) and allele scoring was done using the Saga GT genotyping software (LI-COR Biosciences GmbH, Germany).

The program Structure 2.2 [8] was used to determine population structure and assign accessions to subpopulations. For analysis of population structure and kinship among the accessions selected for this study, 417 of the 460 SNP markers were selected for <25% missing data. We used an ancestry model that allows population admixture, and allele frequencies among population were assumed to be correlated (i.e., allele frequencies were likely to be similar due to shared ancestry or migration). The optimal number of subpopulations was simulated by setting k (number of clusters) from 2 to 10. The length of burn-in period as well as Markov Chain Monte Carlo iterations (MCMC) after burn-in were set to 100,000 for each run and each run was iterated 10 times. An accession was assigned to the subpopulation or group to which it showed the highest probability of membership. Two criteria were used to determine the
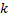
 value which best fits the data. First, the
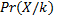
value should be less than or equal to zero, the value of α as a measure of population admixture should remain constant (<0.2) and the cumulative value of Δ for α-factor for next sub-cluster should not decrease. The resulting Q matrix was used for controlling population structure in subsequent association analysis.


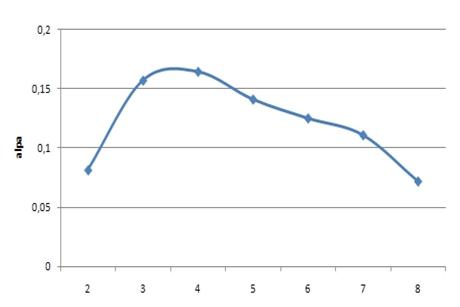


**Figure S1.1:** Cumulative value of Δα for each sub-cluster. Using this graph, the number of populations within the data was determined to be 4 (
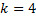
).


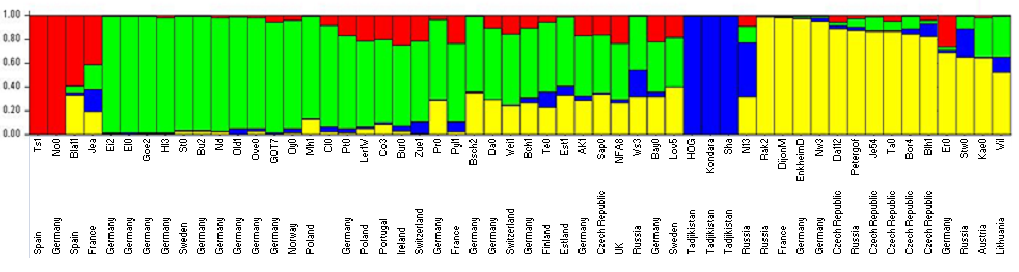


**Figure S1.2:** STRUCTURE analysis of 54 accessions based on 417 SNP markers. Results are shown for
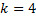
. Each colour represents a different cluster and the proportion of an accession’s bar belonging to a certain colour represents the proportion of the accession belonging to that cluster.

## Analysis of tiling arrays and SFP calling

### SFP identification

Signal intensities obtained from the array hybridisation were analysed using customised software (available upon request) to enable exploration of different normalisation strategies and SFP calling methods. The main functionality of the software is based upon the Tiling-array Analysis Software (TAS) from Affymetrix. To calculate the log fold change, we used the Hodges-Lehmann estimator method. For each probe, the log fold change for all pairs of Col and non-Col chips was calculated and the median was taken.

We utilised the sequence dataset, referred to as the “2010” dataset, from Nordborg et al. [9] to assess the accuracy of our SFP prediction method by estimating the False Discovery Rate (FDR). The 2010 dataset comprises 1,213 fragments of Columbia genomic regions, each approximately 500 nucleotides long, produced through dideoxy-sequencing of 97 accessions. 12 of our accessions were found represented in the 2010 dataset. To allow direct comparison of our results, we converted the SNPs identified in the 2010 dataset to SFPs by mapping them onto the Affymetrix probes. The accuracy was then assessed using Receiver Operator Characteristic (ROC) plots. A False Negative Rate (FNR) was also estimated, using the 2010 data, as the fraction of the remaining sequence-verified polymorphic probes that were not predicted to be polymorphic using our methodology.

We tested several normalisation strategies including invariant normalisation, mode and range normalisation, and quantile normalisation. For invariant normalisation, we identified invariant probes; i.e., probes not found to be polymorphic based on the 2010 dataset, and determined the normalisation shift that aligns the invariant probe signal intensity distributions to those of the Col-0 control hybridisations. Mode and range normalisation adjusts the mode of the signal histogram while ensuring that the lowest and highest values of the distribution are equal to the lowest and highest values of the control distribution. For quantile normalisation, the quantiles of the signal intensity distributions are rendered identical to the control distributions.

We also investigated several methods to improve the FDR of SFP calling. A typical Affymetrix microarray analysis would use the difference between perfect match and mismatch probes as the signal. Here, we also analysed the perfect match probe alone. We also studied the effect of removing outliers reported in the CEL files and substituting an average of the local array area. However, this approach masked the SFPs we seek resulting in poor sensitivity. We also tested the number of forward and reverse arrays required to produce the best result using the initial test cases (Ler-1, Ak-1 and Bch-1). A combinatorial approach using three forward and three reverse arrays, shows that a balance of forward and reverse arrays produces the best result. Naturally, the more arrays used, the better the results. However, the improvement provided by using more arrays peaks at around four. No significant difference between the positions of the SFPs called was detected between forward and reverse arrays. To maximise the number of accessions possible to analyse, we chose to use three reverse arrays and one forward array per accession.

A Linear Discriminant Analysis (LDA) using Statistica 7.1 (http://www.statsoft.com) was performed to compare different SFP calling techniques and identify the best possible combination of predictive variables. It determined that log base 2 (log_2_)-fold change of the perfect match probe alone reflects 98% of the discriminatory information. Among the other variables used in the LDA were the Wilcoxon signed rank test p-value, t-test p-value, GC content (%G+C), RNA-folding free energy (MFE) of the probe as determined by RNAfold [10], normalised absolute intensity signal, difference between observed and expected values as calculated by significance analysis for microarrays (SAM) [11], and the position relative to the centromeres. As a result, we chose to use the log_2_-fold change as the only variable to call SFPs. Based on the ratio of true positives to false positives as determined in the ROC plots, we defined a threshold which discriminated SFP from non-SFP calls. The final combination of parameters used for SFP calling were quantile normalisation, perfect match probes only, no outlier removal, and calling SFPs on the log_2_-fold change. The threshold for calling SFPs was set at a log_2_-fold change of -1.5 which is the equivalent of a 2.8-fold decrease in signal intensity.

### Detection of duplications and deletions

Consecutive stretches of SFPs are indistinguishable from deletions due to the limitations of this method. Such stretches of SFPs are likely due to non-Col-0 alleles in coding genes and thus, we consider stretches of SFPs longer than the average size of an Arabidopsis exon to be deletions giving a threshold of ~350 nucleotides or 10 consecutive SFPs. The gene content for each deletion was extracted and assigned to the functional categories to test for different deletion frequencies. Putative gene duplications were detected by searching for stretches of 10 consecutive probes with a greater than two-fold increase in signal. The functional categories in these regions were also analysed.

# SFP calling

## Array and probe properties

The Affymetrix GeneChip® Arabidopsis Tiling 1.0R array consists 3,039,991 probes which are 25 nucleotides long and are spaced approximately 10 nucleotides apart. (<http://www.affymetrix.com/products/arrays/specific/arab_tiling.affx>). The probes are designed based on the completely sequenced genome of the Arabidopsis Col-0 accession (AGI 2000). Approximately 71.4% of the Col-0 genomic regions are covered by probes on the array.

**Figure S2.1:** Distribution of probe intensities for different GC contents. The bimodal nature of the histogram is intuitively attributed to the signal produced by polymorphic and non-polymorphic probes. However, breaking down the histogram by GC content shows that GC rich probes (blue) have an intrinsically higher intensity and vice versa for GC poor probes (red). To control for the effect of %G+C, control arrays that are hybridised to the reference genome need to be produced.


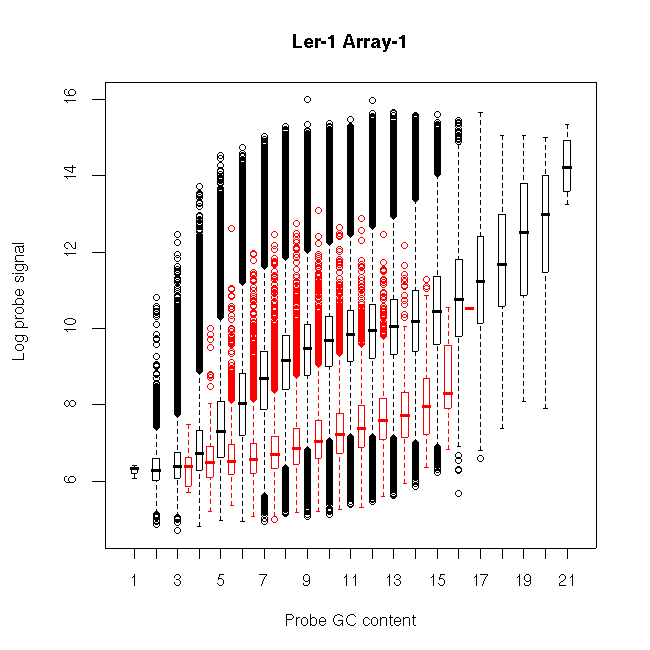


**Figure S2.2:** Effect of GC content on signal intensity. The median signal intensity for non-polymorphic probes is shown in black and increases gradually as the GC content of the probe increases. Polymorphic probes show a significant drop in signal in the %G+C range of 5-15. This figure is a typical profile for the tiling array chips we analysed. By using probes with a %G+C between 6 and 15, it may be possible to improve the FDR at the expense of the FNR.


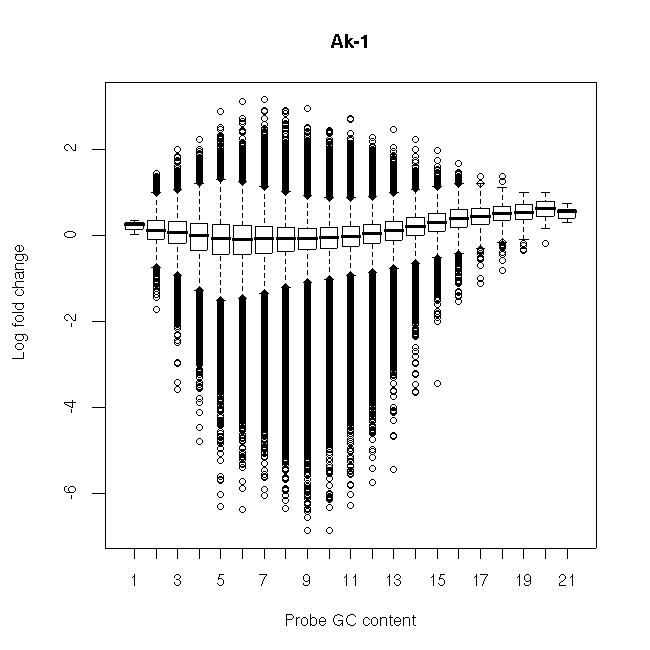


**Figure S2.3:** Effect of GC content on fold change. The median fold change remains relatively stable across probes with differing GC content. This figure is a typical profile for the tiling array chips we used and indicates that no normalisation is needed for %G+C.


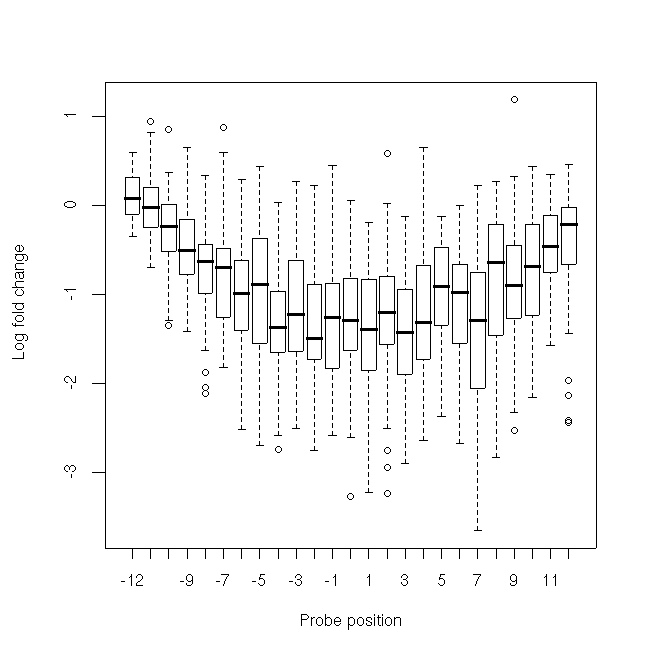


**Figure S2.4:** Effect of SNP position in the probe on probe signal. SNPs which are located toward the middle of the probe peturb the signal more from the Col-0 reference signal than SNPs which are located toward the edges. On average, SNPs located on the very edge of a probe barely perturb the signal at all.


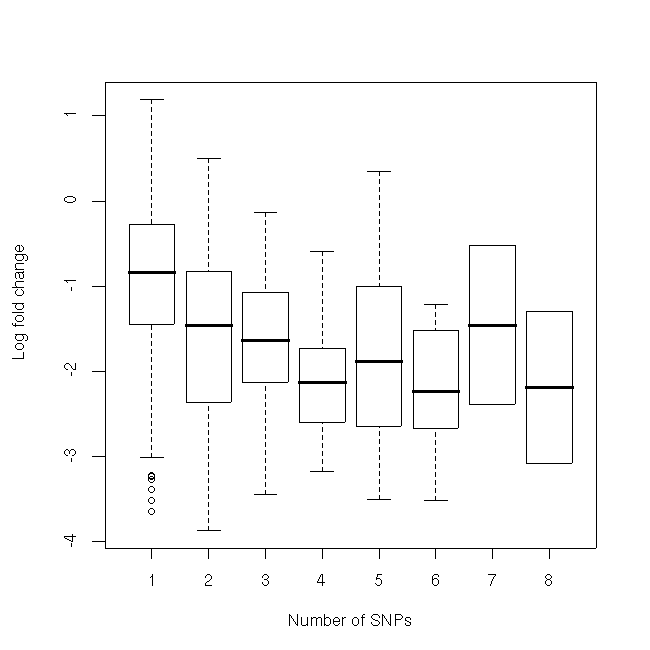


**Figure S2.5:** Influence of number of SNPs in the probe on probe signal. We observed that the more SNPs that occur in a probe, the lower the signal.

## SFP detection


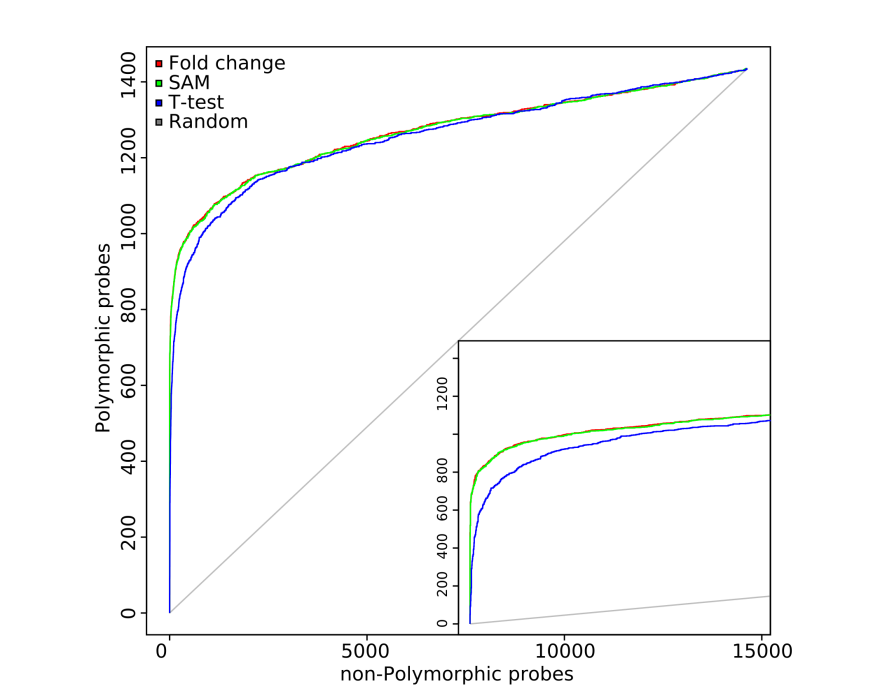


**Figure S2.7:** ROC plot for Landsberg accession. We measured the performance of the 23 accessions that have been previously genotyped by sequencing using ROC plots. As there are almost 10 times as many non-polymorphic probes as there are polymorphic probes, the first part of the curve is shown with equal x and y scales in the inset. This shows that any increase of the curve in the y-axis in the larger figure translates to a very large increase in the y-axis in the inset.

| Accession | FDR(%) | FNR(%) |
| --- | --- | --- |
| **Bay-0** | 12.0 | 60.5 |
| **Bor-4** | 16.0 | 66.8 |
| **Bur-0** | 4.5 | 64.1 |
| **Ei-2** | 3.4 | 70.9 |
| **Est-1** | 8.5 | 68.0 |
| **Got-7** | 3.5 | 66.2 |
| **Ler-1** | 0.0 | 66.2 |
| **Lov-5** | 2.7 | 72.7 |
| **NFA-8** | 6.1 | 65.0 |
| **Oy-0** | 2.2 | 66.4 |
| **Shakdara** | 3.5 | 67.7 |
| **Ts-1** | 4.4 | 66.2 |

**Table S2.1:** False Discovery Rates (FDR) and False Negative Rates (FNR) at a threshold of -1.5. Based on the 12 accession which were in common with the 2010 dataset, we are able estimate the accuracy of the SFPs that we predict. This ranges from 1 false positive for every 202 true positives to 1 false positive for every 16 true positives. The FNR is an estimate of how many of the probes we did not predict to be polymorphic that should have been.

| Acc | SFPs | Dels | Dups | Acc | SFPs | Dels | Dups | Acc | SFPs | Dels | Dups |
| --- | --- | --- | --- | --- | --- | --- | --- | --- | --- | --- | --- |
| **Ak-1** | 163661 | 416 | 0 | **Est-1** | 141812 | 272 | 1 | **Ove-0** | 151935 | 300 | 1 |
| **Bay-0** | 174900 | 578 | 0 | **Goe-2** | 133002 | 248 | 3 | **Oy-0** | 162220 | 486 | 3 |
| **Bch-1** | 165191 | 269 | 2 | **Got-7** | 171876 | 469 | 9 | **Petergof** | 204383 | 617 | 2 |
| **Bla-11** | 112004 | 321 | 3 | **H-O-G** | 231383 | 580 | 0 | **Pr-0** | 155578 | 526 | 0 |
| **Blh-1** | 172571 | 377 | 0 | **Hl-3** | 130467 | 191 | 3 | **Pt-0** | 158246 | 526 | 0 |
| **Bor-4** | 158736 | 358 | 1 | **Je54** | 150293 | 296 | 2 | **Pyl-1** | 161417 | 351 | 0 |
| **Bsch-2** | 166153 | 465 | 1 | **Jea** | 186323 | 442 | 3 | **Rak-2** | 167034 | 260 | 0 |
| **Bu-2** | 150517 | 536 | 0 | **Kae-0** | 159489 | 220 | 0 | **Sap-0** | 156415 | 444 | 3 |
| **Bur-0** | 176397 | 447 | 1 | **Kondora** | 175667 | 394 | 0 | **Shakdara** | 191005 | 399 | 0 |
| **Cl-0** | 167073 | 513 | 0 | **Ler-1** | 180921 | 536 | 0 | **St-0** | 155048 | 495 | 2 |
| **Co-3** | 111259 | 110 | 3 | **Lov-5** | 147539 | 341 | 5 | **Stw-0** | 159813 | 452 | 0 |
| **Da-0** | 162936 | 423 | 2 | **Mh-1** | 140159 | 233 | 1 | **Ta-0** | 152939 | 440 | 1 |
| **Dal-12** | 178588 | 789 | 2 | **N13** | 133162 | 116 | 0 | **Te-0** | 172879 | 402 | 0 |
| **Dijon-M** | 160808 | 272 | 0 | **NFA-8** | 172684 | 553 | 3 | **Ts-1** | 177947 | 500 | 0 |
| **Ei-2** | 157558 | 265 | 1 | **Nd** | 157468 | 686 | 1 | **Wei-1** | 181461 | 262 | 1 |
| **El-0** | 155938 | 619 | 2 | **No-0** | 168235 | 375 | 1 | **Wil** | 206157 | 141 | 0 |
| **Enkheim-D** | 160299 | 412 | 1 | **Nw-3** | 137873 | 400 | 3 | **Ws-3** | 148115 | 244 | 1 |
| **Er-0** | 161670 | 254 | 2 | **Old-1** | 159995 | 374 | 2 | **Zue-1** | 163459 | 307 | 0 |
|  |  |  |  | **Col-0** | 1560 | 2 | 0 |  |  |  |  |

**Table S2.2:** Number of SFPs called per accession. The number of predicted SFPs per accession is listed in ascending order. As Col-0 is the reference genotype, we expect a low number of SFPs to be called. In comparison with the other genotypes, we observe a considerably lower number of SFPs, deletions and duplications.

| **Accession** | **Predicted SFPs** | **Overlap** | **Perlegen SFPs** |
| --- | --- | --- | --- |
| **Bay-0** | 174900 | 57565 | 119306 |
| **Bor-4** | 158736 | 50567 | 120103 |
| **Bur-0** | 176397 | 62023 | 138755 |
| **Est-1** | 141812 | 38666 | 109912 |
| **Got-7** | 171876 | 51249 | 116320 |
| **Ler-1** | 180921 | 53424 | 125025 |
| **Lov-5** | 147539 | 40906 | 129511 |
| **NFA-8** | 172684 | 55323 | 123765 |
| **Shakdara** | 191005 | 48076 | 124864 |
| **Ts-1** | 177947 | 50655 | 124327 |

**Table S2.3:** Overlap with Perlegen dataset [12]. For each of the accessions common to our as well as the Perlegen data, we show how many polymorphisms are uniquely called by our method (Predicted SFPs), how many are uniquely called by the Perlegen dataset (Perlegen SFPs) and how many are shared (Overlap). For this comparison, only sequences represented by probes on the Arabidopsis Tiling 1.0 array were considered (sequence polymorphisms in the Perlegen data set, that lie in the 10 bp gaps between the tiling array probes were not take into account). On average we predict 60,000 previously unidentified SFPs per accession.

## Genetic distance between accessions based on SFP markers


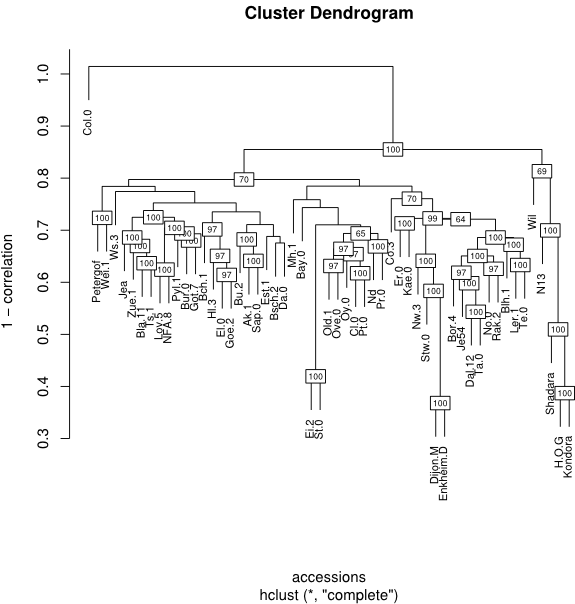


**Figure S2.8:** This tree shows the phylogenetic relationship of 54 accessions using Col-0 as an outlier. It is based on the SFP genotypes and was computed by using the R-script of Borevitz et al. 2007 where the distance between two accessions was computed by 1 minus their pairwise correlation.

# Polymorphism annotation

|  | Family | Observed | Expected | Paired T-test | Further annotation |
| --- | --- | --- | --- | --- | --- |
| Over-represented | Disease resistance (NBS-LRR class) | 1693 | 270.1 | 1.35e-41 | Defense related |
|  | DC1 domain-containing | 411.5 | 119.7 | 3.88e-34 | Zinc binding |
|  | ATP binding | 222 | 133.5 | 4.95e-27 |  |
|  | Transmembrane receptor | 122 | 25.2 | 1.18e-26 | Signalling |
|  | Anthranilate synthase | 57 | 13.2 | 1.00e-22 | Defense related |
|  | Thioredoxin | 25.5 | 11.1 | 1.84e-22 | Signalling |
|  | Leucine-rich repeat kinase | 312.5 | 70.3 | 4.74e-19 | Signalling |
|  | F-box | 714.5 | 590.9 | 8.02e-19 | Defense related |
|  | Terpene synthesis | 82 | 30.9 | 8.16e-19 | Defense related |
|  | Cytoplasmic ribosomal | 188 | 150.4 | 2.12e-18 | Translation |
|  | Monooxygenase | 48 | 17.3 | 3.89e-17 | Defense related |
|  | Carbohydrate esterase | 159.5 | 106.6 | 4.71e-17 |  |
|  | Receptor-like kinase | 1098 | 894.6 | 3.82e-16 | Signalling |
|  | 60s ribosomal | 109 | 81.0 | 8.72e-16 |  |
|  | SNARE and associated proteins | 25.5 | 14.1 | 9.33e-16 |  |
|  | Ubiquitin | 50 | 34.8 | 1.18e-14 |  |
|  | Zinc knuckle (CCHC-type) | 51.5 | 26.8 | 3.28e-14 |  |
|  | Kelch repeat-containing | 79.5 | 22.9 | 5.79e-14 |  |
|  | Wall-associated protein kinase | 26 | 20.2 | 1.62e-03 |  |
|  | Chloroplast and mitochondria gene families | 49 | 43.9 | 2.22e-02 |  |
| Under-represented | Calmodulin binding | 91 | 172.6 | 4.14e-41 |  |
|  | AP2-EREBP transcription factor | 29 | 89.6 | 6.00e-41 | Transcription factor |
|  | C2H2 transcription factor | 115 | 229.5 | 1.11e-39 | Transcription factor |
|  | Helicase | 26 | 66.5 | 9.25e-39 |  |
|  | ABC transporter | 179 | 306.5 | 2.47e-38 |  |
|  | bHLH transcription factor | 99 | 163.2 | 3.20e-36 | Transcription factor |
|  | Glycosyl transferase | 272 | 430.8 | 1.84e-33 |  |
|  | MYB transcription factor | 116.5 | 181.1 | 2.37e-33 | Transcription factor |
|  | Acyl lipid metabolism | 610 | 779.7 | 5.28e-33 |  |
|  | Glycoside hydrolase | 278 | 403.1 | 1.24e-31 |  |
|  | RING | 373.5 | 501.3 | 4.92e-30 | Transcription factor |
|  | Organic solute cotransporter | 383.5 | 475.6 | 4.15e-24 |  |
|  | EF-hand containing | 161.5 | 226.1 | 1.13e-21 |  |
|  | Cytochrome p450 | 195 | 234.4 | 4.14e-08 |  |
|  | Subtilisin-like serine protease | 75.5 | 89.5 | 7.42e-07 |  |
|  | MADS-box transcription factor | 88 | 102.6 | 6.91e-06 | Transcription factor |
|  | Eukaryotic translation initiation factor | 94 | 101.7 | 5.14e-03 | Translation |
|  | Calcium dependent protein kinase | 52 | 58.6 | 2.06e-02 |  |
|  | WRKY transcription factor | 64.5 | 73.2 | 0.223 | Transcription factor |
|  | Plant cell wall biosynthesis | 55 | 91.8 | 0.503 |  |

**Table S3.1:** Frequency of SFPs in gene families. From a larger list of annotation, 40 gene families were chosen based on categories previously analysed in a similar manner and the gene families available from TAIR. For each gene category, the total number of observed polymorphisms is compared to the expected amount assuming an even distribution of SFPs. Previously analysed families are highlighted in blue.

|  | Family | Observed | Expected | Paired T-test | Further annotation |
| --- | --- | --- | --- | --- | --- |
| Over-represented | Anthranilate synthase | 1802.3 | 55.4 | 2.28e-16 | Defense related |
|  | Disease resistance (NBS-LRR class) | 15943.3 | 1134.2 | 4.07e-09 | Defense related |
|  | Kelch repeat-containing | 2336.8 | 96.0 | 1.43e-06 |  |
|  | DC1 domain-containing | 1994.1 | 502.7 | 1.83e-06 | Zinc-binding |
|  | Leucine-rich repeat kinase | 1936.9 | 295.4 | 0.000118 | Signalling |
|  | Carbohydrate esterase | 1952.4 | 447.7 | 0.000691 |  |
|  | Transmembrane receptor | 1159.2 | 105.8 | 0.000909 | Signalling |
|  | ATP binding | 1475.8 | 560.4 | 0.006088 |  |
|  | Plant cell wall biosynthesis | 2752.6 | 385.3 | 0.040772 | Plant cell wall biosynthesis |
|  | Wall-associated protein kinase | 539.9 | 84.8 | 0.097631 | Signalling |
|  | Ubiquitin | 247.6 | 146.3 | 0.169359 |  |
|  | Terpene synthesis | 275.5 | 129.9 | 0.254126 | Defense related |
|  | Receptor-like kinase | 4436.2 | 3756.3 | 0.257614 | Signalling |
|  | Thioredoxin | 70.4 | 46.5 | 0.413717 | Signalling |
|  | Monooxygenase | 108.0 | 72.5 | 0.634048 | Defense related |
|  | F-box | 2583.1 | 2481.4 | 0.779008 | Defense related |
| Under-represented | Calmodulin binding | 0.0 | 724.6 | 1.35e-18 | Flowering |
|  | Helicase | 0.0 | 279.1 | 1.35e-18 |  |
|  | WRKY transcription factor | 0.0 | 307.6 | 1.35e-18 | Transcription factor |
|  | Calcium dependent protein kinase | 0.0 | 246.0 | 1.35e-18 | Signalling |
|  | SNARE and associated proteins | 0.0 | 59.2 | 1.35e-18 |  |
|  | MYB transcription factor | 66.8 | 760.5 | 1.91e-18 | Transcription factor |
|  | Organic solute cotransporter | 64.1 | 1997.2 | 2.78e-17 |  |
|  | Acyl lipid metabolism | 446.1 | 3273.9 | 4.70e-16 |  |
|  | Glycosyl transferase | 120.2 | 1808.8 | 2.99e-15 |  |
|  | Glycoside hydrolase | 168.7 | 1692.5 | 1.70e-12 |  |
|  | Cytochrome p450 | 134.1 | 984.1 | 1.50e-11 |  |
|  | AP2-EREBP transcription factor | 60.5 | 376.4 | 3.70e-10 | Transcription factor |
|  | Cytoplasmic ribosomal | 132.0 | 631.7 | 2.98e-08 | Translation |
|  | bHLH transcription factor | 131.2 | 685.2 | 4.71e-08 | Transcription factor |
|  | ABC transporter | 291.6 | 1286.8 | 3.02e-07 |  |
|  | Subtilisin-like serine protease | 56.5 | 375.7 | 5.96e-06 |  |
|  | EF-hand containing | 178.3 | 949.3 | 0.000128 |  |
|  | C2H2 transcription factor | 266.9 | 963.6 | 0.000862 | Transcription factor |
|  | 60s ribosomal | 132.0 | 340.3 | 0.003262 | Translation |
|  | RING | 1017.9 | 2104.8 | 0.005953 | Transcription factor |
|  | Chloroplast and mitochondria gene families | 55.4 | 184.2 | 0.01957 |  |
|  | Eukaryotic translation initiation factor | 182.6 | 427.1 | 0.025714 | Transcription factor |
|  | MADS-box transcription factor | 336.9 | 430.9 | 0.518499 | Transcription factor |
|  | Zinc knuckle (CCHC-type) | 86.4 | 112.5 | 0.614758 |  |

**Table S3.2:** Frequency of deletions in gene families. The number of deleted nucleotides per family was compared to the expected number assuming an even distribution. Similar to SFPs, defense related and signalling families tend to be enriched in SFPs while transcription factors are depleted. Although SNARE, cytoplasmic ribosomal and zinc-knuckle genes are enriched in SFPs they are depleted in deletions suggesting that the function of these genes families is critical but malleable. Gene families from previous studies are highlighted in blue.

|  | **Family** | **Observed** | **Expected** | **T-test rel** | **Further annotation** |
| --- | --- | --- | --- | --- | --- |
| Over-represented | MADS-box transcription factor | 2879.2 | 116.9 | 0.002172 | Transcription factor |
|  | Glycoside hydrolase | 941.4 | 459.1 | 0.038232 |  |
|  | Disease resistance (NBS-LRR class) | 1074.8 | 307.7 | 0.152303 | Defense related |
|  | F-box | 804.3 | 673.1 | 0.417792 | Defense related |
|  | DC1 domain-containing | 235.9 | 136.4 | 0.463579 | Zinc binding |
|  | Monooxygenase | 61.6 | 19.7 | 0.498792 | Defense related |
|  | Terpene synthesis | 107.0 | 35.2 | 0.504861 | Defense related |
|  | C2H2 transcription factor | 414.1 | 261.4 | 0.655794 | Transcription factor |
| Under-represented | Thioredoxin | 0.0 | 12.6 | 4.76e-16 | Signalling |
|  | Organic solute cotransporter | 0.0 | 541.7 | 4.76e-16 |  |
|  | Carbohydrate esterase | 0.0 | 121.4 | 4.76e-16 |  |
|  | MYB transcription factor | 0.0 | 206.3 | 4.76e-16 | Transcription factor |
|  | Wall-associated protein kinase | 0.0 | 23.0 | 4.76e-16 | Signalling |
|  | Cytoplasmic ribosomal | 0.0 | 171.3 | 4.76e-16 | Translation |
|  | Kelch repeat-containing | 0.0 | 26.0 | 4.76e-16 |  |
|  | WRKY transcription factor | 0.0 | 83.4 | 4.76e-16 | Transcription factor |
|  | EF-hand containing | 0.0 | 257.5 | 4.76e-16 |  |
|  | bHLH transcription factor | 0.0 | 185.9 | 4.76e-16 | Transcription factor |
|  | Transmembrane receptor | 0.0 | 28.7 | 4.76e-16 | Signalling |
|  | 60s ribosomal | 0.0 | 92.3 | 4.76e-16 | Translation |
|  | Calmodulin binding | 0.0 | 196.6 | 4.76e-16 | Flowering |
|  | Anthranilate synthase | 0.0 | 15.0 | 4.76e-16 | Defense related |
|  | ABC transporter | 0.0 | 349.0 | 4.76e-16 |  |
|  | Ubiquitin | 0.0 | 39.7 | 4.76e-16 |  |
|  | Cytochrome p450 | 0.0 | 266.9 | 4.76e-16 |  |
|  | Helicase | 0.0 | 75.7 | 4.76e-16 |  |
|  | Acyl lipid metabolism | 0.0 | 888.0 | 4.76e-16 |  |
|  | Chloroplast and mitochondria gene families | 0.0 | 50.0 | 4.76e-16 |  |
|  | AP2-EREBP transcription factor | 0.0 | 102.1 | 4.76e-16 | Transcription factor |
|  | SNARE and associated proteins | 0.0 | 16.1 | 4.76e-16 |  |
|  | Zinc knuckle (CCHC-type) | 0.0 | 30.5 | 4.76e-16 |  |
|  | Subtilisin-like serine protease | 0.0 | 101.9 | 4.76e-16 |  |
|  | Calcium dependent protein kinase | 0.0 | 66.7 | 4.76e-16 | Signalling |
|  | Plant cell wall biosynthesis | 0.0 | 104.5 | 4.76e-16 | Plant cell wall biosynthesis |
|  | Eukaryotic translation initiation factor | 30.0 | 115.8 | 0.007716 | Translation |
|  | Receptor-like kinase | 724.1 | 1018.9 | 0.332623 |  |
|  | Glycosyl transferase | 382.0 | 490.6 | 0.674231 |  |
|  | ATP binding | 143.0 | 152.0 | 0.908531 |  |
|  | Leucine-rich repeat kinase | 72.6 | 80.1 | 0.918491 | Signalling |
|  | RING | 567.8 | 570.9 | 0.99244 | Transcription factor |

**Table S3.3:** Frequency of duplications in gene families. Most gene families have no duplicated regions within their members and only MADS-box transcription factors and glycoside hydrolase genes have significant enrichment in duplications. Gene families from previous studies are highlighted in blue.

# Metabolic gene/phenotype association

| **Trait** | **Gene** | **Pathways** | **p-val** | **Col** | **non-Col** |
| --- | --- | --- | --- | --- | --- |
| **Fresh weight** | AT1G15120 | aerobic respiration | 1.26e-04 | 236.13 | 363.70 |
| **Fresh weight** | AT1G51680 | flavonoid biosynthesis  phenylpropanoid biosynthesis  scopoletin biosynthesis  simple coumarins biosynthesis | 3.85e-04 | 223.04 | 270.18 |
| **Fresh weight** | AT1G76680 | jasmonic acid biosynthesis | 9.01e-04 | 237.19 | 346.97 |
| **Fresh weight** | AT2G21770 | cellulose biosynthesis | 8.18e-04 | 234.80 | 284.71 |
| **Fresh weight** | AT2G32620 | cellulose biosynthesis | 1.35e-04 | 234.85 | 309.28 |
| **Fresh weight** | AT2G35690 | fatty acid β-oxidation I (saturated) | 3.01e-04 | 220.63 | 284.94 |
| **Fresh weight** | AT3G05630 | choline biosynthesis III  phospholipases | 9.20e-04 | 252.05 | 169.38 |
| **Fresh weight** | AT3G25810 | monoterpene biosynthesis | 9.01e-04 | 237.19 | 346.97 |
| **Fresh weight** | AT4G14210 | *trans*-lycopene biosynthesis | 7.61e-05 | 234.61 | 349.25 |
| **Fresh weight** | AT5G11110 | sucrose biosynthesis | 9.01e-04 | 237.19 | 346.97 |
| **Fresh weight** | AT5G15950 | spermidine biosynthesis  spermine biosynthesis | 9.01e-04 | 237.19 | 346.97 |
| **Fresh weight** | AT5G28020 | cysteine biosynthesis | 9.08e-05 | 236.50 | 327.55 |
| **Fresh weight** | AT5G37180 | UDP-glucose biosynthesis (from sucrose)  UDP-sugars interconversion  galactose degradation I  sucrose biosynthesis  sucrose degradation | 4.41e-04 | 238.63 | 324.40 |
| **Fresh weight** | AT5G41890 | triacylglycerol degradation | 3.21e-04 | 232.43 | 313.47 |
| **Fresh weight** | AT5G43940 | formaldehyde oxidation (glutathione-dependent) | 9.55e-04 | 226.09 | 270.31 |
| **Total protein** | AT1G02460 | homogalacturonan degradation | 9.14e-04 | 18.15 | 21.50 |
| **Total protein** | AT1G33320 | homocysteine and cysteine interconversion  methionine biosynthesis | 4.81e-04 | 19.03 | 17.60 |
| **Total protein** | AT1G54040 | glucosinolate breakdown | 7.67e-05 | 18.13 | 22.05 |
| **Total protein** | AT1G65520 | fatty acid β-oxidation II (unsaturated, even number) | 8.15e-04 | 17.67 | 18.77 |
| **Total protein** | AT2G16500 | putrescine biosynthesis by agmatinase putrescine biosynthesis via *N*-carbamoylputrescine | 7.37e-04 | 18.08 | 20.60 |
| **Total protein** | AT3G45140 | 13-LOX and 13-HPL pathway  jasmonic acid biosynthesis | 4.17e-04 | 18.11 | 20.97 |
| **Total protein** | AT3G48080 | triacylglycerol degradation | 9.34e-04 | 18.01 | 19.96 |
| **Total protein** | AT5G03290 | TCA cycle | 1.25e-04 | 18.18 | 23.60 |
| **Total protein** | AT5G27600 | acyl-CoA synthetase pathway  fatty acid β-oxidation I (saturated)  linoleate biosynthesis | 6.21e-04 | 18.15 | 21.55 |
| **Total amino acids** | AT1G02460 | homogalacturonan degradation | 5.24e-04 | 18.95 | 24.75 |
| **Total amino acids** | AT1G10640 | homogalacturonan degradation | 1.99e-05 | 18.84 | 24.63 |
| **Total amino acids** | AT1G17890 | GDP-L-fucose biosynthesis I (from GDP-D-mannose) | 8.61e-05 | 18.82 | 23.38 |
| **Total amino acids** | AT1G18500 | leucine biosynthesis | 1.11e-04 | 18.88 | 24.00 |
| **Total amino acids** | AT1G20490 | flavonoid biosynthesis  phenylpropanoid biosynthesis  scopoletin biosynthesis  simple coumarins biosynthesis | 6.61e-04 | 18.58 | 21.32 |
| **Total amino acids** | AT1G54040 | glucosinolate breakdown | 3.87e-06 | 18.90 | 26.05 |
| **Total amino acids** | AT1G67550 | urea degradation | 1.98e-05 | 22.49 | 18.65 |
| **Total amino acids** | AT1G68020 | trehalose biosynthesis | 2.04e-04 | 18.84 | 23.20 |
| **Total amino acids** | AT1G68460 | *trans*-zeatin biosynthesis | 5.68e-05 | 18.87 | 24.20 |
| **Total amino acids** | AT2G01918 | photosynthesis  photosynthesis, light reaction | 5.45e-04 | 18.86 | 22.98 |
| **Total amino acids** | AT2G26540 | tetrapyrrole biosynthesis | 6.23e-04 | 18.79 | 22.76 |
| **Total amino acids** | AT2G30550 | triacylglycerol degradation | 8.66e-04 | 20.13 | 18.08 |
| **Total amino acids** | AT2G39930 | starch degradation | 1.42e-06 | 18.87 | 26.70 |
| **Total amino acids** | AT2G44520 | geranyldiphosphate biosynthesis  linalool biosynthesis  polyisoprenoid biosynthesis  trans,trans-farnesyl diphosphate biosynthesis | 6.77e-04 | 18.91 | 23.50 |
| **Total amino acids** | AT2G47650 | UDP-D-xylose biosynthesis  UDP-sugars interconversion | 9.90e-05 | 18.81 | 23.48 |
| **Total amino acids** | AT3G02350 | homogalacturonan biosynthesis | 3.17e-04 | 18.89 | 23.80 |
| **Total amino acids** | AT3G02360 | oxidative branch of the pentose phosphate pathway | 3.17e-04 | 18.89 | 23.80 |
| **Total amino acids** | AT3G02570 | GDP-D-mannose biosynthesis  ascorbate biosynthesis I (L-galactose pathway)  mannitol degradation  mannose degradation | 3.59e-04 | 18.86 | 24.27 |
| **Total amino acids** | AT3G03050 | cellulose biosynthesis | 9.32e-04 | 18.81 | 21.93 |
| **Total amino acids** | AT3G06350 | chorismate biosynthesis | 9.75e-04 | 18.89 | 23.80 |
| **Total amino acids** | AT3G13110 | cysteine biosynthesis | 3.17e-04 | 18.89 | 23.80 |
| **Total amino acids** | AT3G14415 | photorespiration | 6.78e-04 | 18.95 | 24.75 |
| **Total amino acids** | AT3G15730 | choline biosynthesis III phospholipases | 2.14e-05 | 18.84 | 24.67 |
| **Total amino acids** | AT3G43190 | UDP-glucose biosynthesis (from sucrose) UDP-sugars interconversion galactose degradation I sucrose biosynthesis sucrose degradation | 7.01e-04 | 18.91 | 23.53 |
| **Total amino acids** | AT3G46970 | starch degradation | 6.62e-05 | 18.92 | 22.28 |
| **Total amino acids** | AT3G47290 | phospholipases | 4.33e-04 | 18.82 | 23.38 |
| **Total amino acids** | AT3G47340 | asparagine biosynthesis I | 7.24e-04 | 18.80 | 22.64 |
| **Total amino acids** | AT3G49700 | ethylene biosynthesis from methionine methionine salvage pathway | 8.08e-04 | 18.95 | 24.75 |
| **Total amino acids** | AT3G55030 | cardiolipin biosynthesis phosphatidylglycerol biosynthesis I (plastid) phosphatidylglycerol biosynthesis II phospholipid biosynthesis | 1.91e-04 | 18.78 | 22.88 |
| **Total amino acids** | AT3G59760 | cysteine biosynthesis | 1.99e-04 | 18.80 | 22.68 |
| **Total amino acids** | AT4G18350 | abscisic acid biosynthesis | 1.63e-05 | 18.89 | 26.35 |
| **Total amino acids** | AT4G21200 | gibberellin inactivation | 1.87e-04 | 18.88 | 23.97 |
| **Total amino acids** | AT4G29010 | fatty acid β-oxidation I (saturated) fatty acid β-oxidation II (unsaturated, even number) lysine degradation II | 4.25e-04 | 19.01 | 20.80 |
| **Total amino acids** | AT4G29570 | (deoxy)ribose phosphate degradation | 4.58e-05 | 18.97 | 21.12 |
| **Total amino acids** | AT5G03290 | TCA cycle | 7.12e-08 | 18.95 | 30.90 |
| **Total amino acids** | AT5G07990 | flavonol biosynthesis leucopelargonidin and leucocyanidin biosynthesis | 6.00e-05 | 18.90 | 23.63 |
| **Total amino acids** | AT5G22300 | IAA biosynthesis I asparagine biosynthesis II | 2.41e-04 | 18.94 | 25.10 |
| **Total amino acids** | AT5G27600 | acyl-CoA synthetase pathway fatty acid β-oxidation I (saturated) linoleate biosynthesis | 4.05e-04 | 18.94 | 25.05 |
| **Total amino acids** | AT5G43780 | sulfate activation for sulfonation sulfate reduction (assimilatory) | 2.85e-05 | 18.76 | 24.10 |
| **Total amino acids** | AT5G62575 | TCA cycle aerobic respiration aerobic respiration -- alternative oxidase pathway | 9.03e-04 | 18.99 | 20.65 |
| **β-alanine** | AT1G09490 | phenylpropanoid biosynthesis | 8.24e-05 | 0.49 | 0.60 |
| **β-alanine** | AT1G53310 | CO_2_ fixation into oxaloacetate superpathway of glyoxylate cycle | 2.71e-04 | 0.47 | 0.72 |
| **β-alanine** | AT1G60140 | trehalose biosynthesis | 6.07e-05 | 0.48 | 0.85 |
| **β-alanine** | AT1G72590 | brassinosteroid biosynthesis | 3.61e-04 | 0.48 | 0.85 |
| **β-alanine** | AT1G77760 | nitrate assimilation pathway | 1.71e-05 | 0.48 | 0.83 |
| **β-alanine** | AT1G78960 | α-amyrin biosynthesis | 8.24e-05 | 0.49 | 0.60 |
| **β-alanine** | AT2G16500 | putrescine biosynthesis by agmatinase putrescine biosynthesis via *N*-carbamoylputrescine | 2.06e-04 | 0.48 | 0.80 |
| **β-alanine** | AT3G11950 | geranyldiphosphate biosynthesis linalool biosynthesis polyisoprenoid biosynthesis trans,trans-farnesyl diphosphate biosynthesis | 1.46e-06 | 0.48 | 0.95 |
| **β-alanine** | AT3G27980 | homogalacturonan degradation | 2.09e-04 | 0.46 | 0.60 |
| **β-alanine** | AT4G11600 | glutathione redox reactions | 1.86e-04 | 0.48 | 0.80 |
| **β-alanine** | AT4G11820 | mevalonate pathway | 1.94e-04 | 0.48 | 0.80 |
| **β-alanine** | AT4G16700 | phosphatidylethanolamine biosynthesis I phospholipid biosynthesis | 8.55e-04 | 0.48 | 0.61 |
| **β-alanine** | AT4G16820 | triacylglycerol degradation | 7.89e-04 | 0.49 | 0.67 |
| **β-alanine** | AT4G24000 | cellulose biosynthesis | 8.51e-04 | 0.48 | 0.70 |
| **β-alanine** | AT4G24650 | *trans*-zeatin biosynthesis | 9.16e-04 | 0.47 | 0.63 |
| **β-alanine** | AT4G34860 | sucrose degradation | 8.60e-04 | 0.48 | 0.73 |
| **β-alanine** | AT5G14970 | β-alanine biosynthesis I | 7.66e-04 | 0.48 | 0.70 |
| **β-alanine** | AT5G17990 | tryptophan biosynthesis | 6.41e-04 | 0.59 | 0.48 |
| **β-alanine** | AT5G38710 | citrulline biosynthesis proline degradation | 3.46e-04 | 0.47 | 0.61 |
| **β-alanine** | AT5G47990 | thalianol and derivatives biosynthesis | 7.76e-04 | 0.48 | 0.73 |
| **Erythritol** | AT1G02810 | homogalacturonan degradation | 9.70e-04 | 0.80 | 1.10 |
| **Erythritol** | AT1G04410 | TCA cycle glyoxylate cycle | 2.32e-04 | 0.80 | 1.03 |
| **Erythritol** | AT1G11870 | tRNA charging pathway | 3.14e-05 | 0.80 | 1.25 |
| **Erythritol** | AT1G16780 | UDP-glucose biosynthesis (from glucose 6-phosphate) | 3.59e-04 | 0.79 | 1.13 |
| **Erythritol** | AT1G42970 | Calvin cycle gluconeogenesis glycolysis I (plant cytosol) glycolysis II (plant plastids) photosynthesis sucrose degradation to ethanol and lactate (anaerobic) | 4.88e-06 | 0.79 | 1.23 |
| **Erythritol** | AT1G47290 | cholesterol biosynthesis | 1.74e-04 | 0.79 | 1.13 |
| **Erythritol** | AT1G48520 | asparaginyl-tRNA^asn^ biosynthesis via transamidation glutaminyl-tRNA^gln^ biosynthesis via transamidation | 7.48e-04 | 0.85 | 0.68 |
| **Erythritol** | AT1G67550 | urea degradation | 7.10e-04 | 0.80 | 1.20 |
| **Erythritol** | AT1G67560 | 13-LOX and 13-HPL pathway jasmonic acid biosynthesis | 8.71e-04 | 0.80 | 1.10 |
| **Erythritol** | AT2G06050 | jasmonic acid biosynthesis | 6.73e-07 | 1.25 | 0.80 |
| **Erythritol** | AT2G17640 | cysteine biosynthesis | 6.81e-04 | 1.10 | 0.80 |
| **Erythritol** | AT2G33160 | homogalacturonan degradation | 4.80e-05 | 0.80 | 1.07 |
| **Erythritol** | AT2G43860 | homogalacturonan degradation | 1.76e-04 | 0.79 | 1.13 |
| **Erythritol** | AT3G19820 | sterol biosynthesis | 8.66e-04 | 0.80 | 1.10 |
| **Erythritol** | AT3G43270 | homogalacturonan degradation | 1.76e-04 | 0.79 | 1.13 |
| **Erythritol** | AT3G47290 | phospholipases | 1.00e-03 | 0.79 | 1.08 |
| **Erythritol** | AT3G47400 | homogalacturonan degradation | 8.36e-04 | 0.80 | 1.20 |
| **Erythritol** | AT3G53520 | UDP-D-xylose biosynthesis UDP-sugars interconversion | 6.73e-07 | 1.25 | 0.80 |
| **Erythritol** | AT3G55360 | brassinosteroid biosynthesis very long chain fatty acid biosynthesis | 7.00e-04 | 0.79 | 1.05 |
| **Erythritol** | AT3G59890 | lysine biosynthesis I | 7.10e-04 | 0.80 | 1.20 |
| **Erythritol** | AT4G08390 | ascorbate glutathione cycle | 1.50e-04 | 0.79 | 1.04 |
| **Erythritol** | AT4G15480 | sinapate ester biosynthesis | 8.57e-04 | 0.80 | 1.20 |
| **Erythritol** | AT4G16760 | fatty acid β-oxidation I (saturated) | 5.82e-04 | 0.80 | 1.20 |
| **Erythritol** | AT4G19010 | flavonoid biosynthesis phenylpropanoid biosynthesis scopoletin biosynthesis simple coumarins biosynthesis | 7.10e-04 | 0.80 | 1.20 |
| **Erythritol** | AT4G23990 | cellulose biosynthesis | 3.14e-05 | 0.80 | 1.25 |
| **Erythritol** | AT5G17330 | glutamate degradation II | 4.80e-05 | 0.80 | 1.07 |
| **Erythritol** | AT5G18640 | triacylglycerol degradation | 6.87e-04 | 0.79 | 1.13 |
| **Erythritol** | AT5G35630 | ammonia assimilation cycle glutamine biosynthesis nitrate assimilation pathway | 6.21e-04 | 0.79 | 1.13 |
| **Erythritol** | AT5G42600 | marneral biosynthesis | 8.36e-04 | 0.80 | 1.20 |
| **Erythritol** | AT5G48220 | tryptophan biosynthesis | 6.18e-04 | 0.87 | 0.73 |
| **Myo-inositol** | AT1G03090 | leucine degradation | 1.08e-04 | 2.24 | 2.83 |
| **Myo-inositol** | AT1G11680 | sterol biosynthesis | 7.10e-04 | 2.23 | 2.80 |
| **Myo-inositol** | AT1G28570 | triacylglycerol degradation | 7.15e-04 | 2.18 | 2.95 |
| **Myo-inositol** | AT1G29410 | tryptophan biosynthesis | 7.71e-06 | 2.23 | 4.20 |
| **Myo-inositol** | AT1G58080 | histidine biosynthesis | 3.89e-04 | 2.16 | 2.65 |
| **Myo-inositol** | AT1G69830 | starch degradation | 8.25e-04 | 2.15 | 2.91 |
| **Myo-inositol** | AT1G76130 | starch degradation | 7.70e-04 | 2.18 | 3.08 |
| **Myo-inositol** | AT2G29690 | tryptophan biosynthesis | 3.78e-04 | 2.24 | 2.58 |
| **Myo-inositol** | AT2G30575 | homogalacturonan biosynthesis | 9.07e-04 | 2.18 | 3.33 |
| **Myo-inositol** | AT2G38650 | homogalacturonan biosynthesis | 2.51e-04 | 2.22 | 2.93 |
| **Myo-inositol** | AT2G43760 | molybdenum cofactor biosynthesis | 3.82e-04 | 2.20 | 3.50 |
| **Myo-inositol** | AT3G25760 | jasmonic acid biosynthesis | 2.60e-05 | 2.19 | 3.67 |
| **Myo-inositol** | AT3G49680 | isoleucine biosynthesis isoleucine degradation leucine biosynthesis leucine degradation pantothenate biosynthesis  valine biosynthesis valine degradation | 6.51e-04 | 2.20 | 2.96 |
| **Myo-inositol** | AT3G62170 | homogalacturonan degradation | 2.24e-05 | 2.18 | 3.35 |
| **Myo-inositol** | AT4G00190 | homogalacturonan degradation | 3.82e-04 | 2.15 | 2.72 |
| **Myo-inositol** | AT4G04610 | sulfate reduction (assimilatory) | 8.98e-04 | 2.05 | 2.64 |
| **Myo-inositol** | AT4G20930 | oxidative branch of the pentose phosphate pathway | 1.00e-03 | 2.18 | 2.97 |
| **Myo-inositol** | AT4G21280 | photosynthesis, light reaction | 1.08e-04 | 2.24 | 2.83 |
| **Myo-inositol** | AT5G28030 | cysteine biosynthesis | 2.89e-04 | 2.20 | 3.18 |
| **Myo-inositol** | AT5G38630 | carbon tetrachloride degradation | 6.60e-04 | 2.61 | 2.10 |
| **Myo-inositol** | AT5G47435 | folate transformations | 2.60e-04 | 2.21 | 3.27 |
| **Myo-inositol** | AT5G47780 | homogalacturonan biosynthesis | 2.12e-05 | 2.17 | 3.50 |
| **Myo-inositol** | AT5G48220 | tryptophan biosynthesis | 4.41e-04 | 2.13 | 2.77 |
| **Myo-inositol** | AT5G49810 | S-methylmethionine cycle | 3.57e-04 | 2.17 | 2.71 |
| **Myo-inositol** | AT5G51690 | aspartate biosynthesis  tyrosine biosynthesis I | 3.97e-04 | 2.52 | 1.96 |
| **Myo-inositol** | AT5G65010 | asparagine biosynthesis I | 1.53e-04 | 2.24 | 2.83 |
| **Starch** | AT1G09940 | tetrapyrrole biosynthesis | 2.09e-04 | 37.07 | 42.15 |
| **Starch** | AT1G79500 | CMP-KDO biosynthesis I (from D-arabinose 5-phosphate) CMP-KDO biosynthesis II (from D-ribulose 5-phosphate) | 6.61e-04 | 42.08 | 38.64 |
| **Starch** | AT2G23420 | pyridine nucleotide cycling (plants) | 8.72e-04 | 39.83 | 43.76 |
| **Starch** | AT2G39930 | starch degradation | 8.49e-04 | 39.74 | 51.90 |
| **Starch** | AT2G44520 | geranyldiphosphate biosynthesis linalool biosynthesis polyisoprenoid biosynthesis  trans,trans-farnesyl diphosphate biosynthesis | 7.73e-04 | 39.68 | 48.70 |
| **Starch** | AT3G15730 | choline biosynthesis III phospholipases | 9.99e-04 | 39.64 | 49.37 |
| **Starch** | AT3G27980 | homogalacturonan degradation | 1.15e-04 | 42.02 | 37.54 |
| **Starch** | AT3G45140 | 13-LOX and 13-HPL pathway jasmonic acid biosynthesis | 1.44e-04 | 39.47 | 52.07 |
| **Starch** | AT4G29210 | γ-glutamyl cycle glutathione degradation | 2.48e-04 | 39.70 | 52.85 |
| **Starch** | AT5G04040 | triacylglycerol degradation | 9.96e-04 | 39.48 | 51.90 |
| **Starch** | AT5G43940 | formaldehyde oxidation (glutathione-dependent) | 7.40e-04 | 41.83 | 37.82 |
| **Starch** | AT5G50375 | sterol biosynthesis | 8.90e-04 | 38.80 | 44.28 |
| **Sucrose** | AT1G23800 | fatty acid α-oxidation fatty acid ω-oxidation | 5.86e-04 | 3.99 | 4.41 |
| **Sucrose** | AT1G53990 | triacylglycerol degradation | 9.54e-04 | 4.04 | 4.41 |
| **Sucrose** | AT1G54040 | glucosinolate breakdown | 6.00e-04 | 4.08 | 4.78 |
| **Sucrose** | AT2G05990 | fatty acid elongation -- saturated | 1.04e-04 | 4.40 | 3.97 |
| **Sucrose** | AT3G47340 | asparagine biosynthesis I | 9.81e-05 | 4.08 | 4.84 |
| **Sucrose** | AT5G05870 | cytokinins 7-*N*-glucoside biosynthesis cytokinins 9-*N*-glucoside biosynthesis | 7.45e-04 | 4.03 | 4.43 |
| **Sucrose** | AT5G35790 | oxidative branch of the pentose phosphate pathway | 4.62e-04 | 4.24 | 3.59 |
| **Sucrose** | AT5G38710 | citrulline biosynthesis proline degradation | 1.00e-03 | 4.03 | 4.46 |
| **Sucrose** | AT5G43710 | 2-*O*-α-mannosyl-D-glycerate degradation | 1.15e-04 | 4.51 | 4.01 |
| **Sucrose** | AT5G43940 | formaldehyde oxidation (glutathione-dependent) | 2.53e-04 | 4.30 | 3.97 |
| **Threonic acid** | AT1G07890 | ascorbate glutathione cycle | 2.75e-04 | 1.13 | 2.07 |
| **Threonic acid** | AT1G08550 | antheraxanthin and violaxanthin biosynthesis superpathway of carotenoid biosynthesis xanthophyll cycle | 4.18e-04 | 1.14 | 1.97 |
| **Threonic acid** | AT1G20575 | dolichyl-diphosphooligosaccharide biosynthesis | 1.98e-05 | 1.13 | 2.17 |
| **Threonic acid** | AT1G30380 | photosynthesis, light reaction | 3.28e-04 | 1.13 | 2.17 |
| **Threonic acid** | AT1G47260 | NAD/NADH phosphorylation and dephosphorylation aerobic respiration -- alternative oxidase pathway | 1.57e-04 | 1.13 | 2.13 |
| **Threonic acid** | AT1G48520 | asparaginyl-tRNA^asn^ biosynthesis via transamidation glutaminyl-tRNA^gln^ biosynthesis via transamidation | 1.40e-05 | 1.12 | 2.30 |
| **Threonic acid** | AT1G55020 | 13-LOX and 13-HPL pathway jasmonic acid biosynthesis | 6.37e-04 | 1.12 | 1.84 |
| **Threonic acid** | AT1G56190 | Calvin cycle gluconeogenesis glycolysis I (plant cytosol) glycolysis II (plant plastids) photosynthesis sucrose degradation to ethanol and lactate (anaerobic) | 4.20e-05 | 1.12 | 2.20 |
| **Threonic acid** | AT1G56600 | stachyose biosynthesis | 5.03e-05 | 1.10 | 1.94 |
| **Threonic acid** | AT1G56710 | homogalacturonan degradation | 6.42e-04 | 1.08 | 1.67 |
| **Threonic acid** | AT2G24270 | glycolysis I (plant cytosol) | 4.51e-04 | 1.17 | 1.43 |
| **Threonic acid** | AT2G34890 | de novo biosynthesis of pyrimidine ribonucleotides pyrimidine nucleotide metabolism | 6.61e-04 | 1.10 | 1.76 |
| **Threonic acid** | AT2G39290 | cardiolipin biosynthesis phosphatidylglycerol biosynthesis I (plastid) phosphatidylglycerol biosynthesis II phospholipid biosynthesis | 1.17e-05 | 1.14 | 2.40 |
| **Threonic acid** | AT2G47030 | homogalacturonan degradation | 1.59e-05 | 1.07 | 1.91 |
| **Threonic acid** | AT2G47040 | homogalacturonan degradation | 1.59e-05 | 1.07 | 1.91 |
| **Threonic acid** | AT3G05610 | homogalacturonan degradation | 1.77e-04 | 1.13 | 2.17 |
| **Threonic acid** | AT3G11670 | glycolipid biosynthesis | 7.59e-04 | 1.13 | 2.13 |
| **Threonic acid** | AT3G14300 | homogalacturonan degradation | 2.75e-04 | 1.13 | 2.07 |
| **Threonic acid** | AT3G45140 | 13-LOX and 13-HPL pathway jasmonic acid biosynthesis | 9.05e-04 | 1.10 | 1.80 |
| **Threonic acid** | AT3G52990 | Rubisco shunt glycolysis I (plant cytosol) glycolysis II (plant plastids) sucrose degradation to ethanol and lactate (anaerobic) | 5.78e-05 | 1.12 | 2.00 |
| **Threonic acid** | AT3G60730 | homogalacturonan degradation | 9.54e-04 | 1.11 | 1.78 |
| **Threonic acid** | AT3G61130 | homogalacturonan biosynthesis | 8.24e-04 | 1.13 | 1.72 |
| **Threonic acid** | AT4G00400 | CDP-diacylglycerol biosynthesis I CDP-diacylglycerol biosynthesis II phosphatidylglycerol biosynthesis I (plastid) phosphatidylglycerol biosynthesis II phospholipid biosynthesis triacylglycerol biosynthesis | 5.72e-04 | 1.13 | 1.85 |
| **Threonic acid** | AT4G05160 | flavonoid biosynthesis phenylpropanoid biosynthesis scopoletin biosynthesis simple coumarins biosynthesis | 3.71e-04 | 1.13 | 2.17 |
| **Threonic acid** | AT4G12110 | cholesterol biosynthesis | 6.66e-04 | 1.08 | 1.58 |
| **Threonic acid** | AT4G15210 | starch degradation | 8.96e-05 | 1.11 | 1.86 |
| **Threonic acid** | AT4G15490 | sinapate ester biosynthesis | 6.76e-05 | 1.13 | 1.85 |
| **Threonic acid** | AT4G16310 | β-alanine biosynthesis I | 5.64e-05 | 1.12 | 2.20 |
| **Threonic acid** | AT5G09870 | cellulose biosynthesis | 3.59e-05 | 1.11 | 2.08 |
| **Threonic acid** | AT5G20040 | *cis*-zeatin biosynthesis | 5.90e-04 | 1.08 | 1.70 |
| **Threonic acid** | AT5G24220 | triacylglycerol degradation | 1.81e-05 | 1.08 | 1.74 |
| **Threonic acid** | AT5G38830 | tRNA charging pathway | 1.55e-04 | 1.14 | 2.30 |
| **Threonic acid** | AT5G45930 | chlorophyllide *a* biosynthesis | 3.37e-04 | 1.14 | 2.35 |
| **Threonic acid** | AT5G48010 | thalianol and derivatives biosynthesis | 6.43e-04 | 1.13 | 2.07 |
| **Threonic acid** | AT5G50160 | Fe(III)-reduction and Fe(II) transport | 4.44e-05 | 2.00 | 1.15 |
| **Threonic acid** | AT5G53970 | 4-hydroxyphenylpyruvate biosynthesis  tyrosine degradation | 1.01e-06 | 1.13 | 2.65 |

**Table S4.1:** Measured phenotypic traits and the significantly associated genes. Many of the associated metabolic pathways are directly related to the phenotypic trait.

| **Case** | **Pathway** | **p-value** | **Average pathway F-score** | **Average metabolic**  **F-score** |
| --- | --- | --- | --- | --- |
| **Fresh weight** | simple coumarins biosynthesis | 7.66E-03 | 4.82 | 2.89 |
| **Total protein** | simple coumarins biosynthesis | 4.70E-03 | 4.61 | 2.85 |
| **Total protein** | aerobic respiration | 7.47E-03 | 1.88 | 2.89 |
| **Total protein** | sinapate ester biosynthesis | 7.66E-03 | 5.58 | 2.85 |
| **Total protein** | tetrahydrofolate biosynthesis | 7.70E-03 | 1.11 | 2.88 |
| **Erythritol** | aerobic respiration | 2.09E-03 | 1.33 | 2.68 |
| **Starch** | aerobic respiration | 3.28E-03 | 1.48 | 2.64 |
| **Starch** | ammonia assimilation cycle | 9.71E-03 | 4.89 | 2.60 |
| **Sucrose** | simple coumarins biosynthesis | 9.71E-03 | 4.12 | 2.58 |
| **Sucrose** | aerobic respiration | 7.47E-03 | 1.66 | 2.62 |
| **Sucrose** | acetyl-CoA biosynthesis (from pyruvate) | 7.66E-03 | 1.23 | 2.61 |
| **Sucrose** | tyrosine biosynthesis II | 7.70E-03 | 5.58 | 2.59 |
| **Myo-inositol** | simple coumarins biosynthesis | 2.09E-03 | 5.03 | 2.73 |
| **Myo-inositol** | flavonoid biosynthesis | 3.28E-03 | 4.38 | 2.73 |
| **Myo-inositol** | scopoletin biosynthesis | 4.62E-03 | 4.69 | 2.73 |
| **Myo-inositol** | ascorbate glutathione cycle | 7.47E-03 | 1.10 | 2.77 |
| **Threonic acid** | aerobic respiration | 4.62E-03 | 1.93 | 2.93 |
| **Threonic acid** | photosynthesis, light reaction | 4.59E-03 | 1.60 | 2.91 |

**Table S4.2:** Over-represented pathways. For each phenotype, twelve selected pathways of interest were tested for overrepresentation using the Mann-Whitney U to test F-scores for SFP sites within genes belonging to a given pathway against all other metabolic SFP sites. Seven phenotypes were found to have F-scores significantly higher or lower than the rest of the metabolic genes. Only fifteen pathways were chosen to reduce the multiple testing problem. The reported p-value has been corrected using Benjamini-Hochberg correction. If the F-score for the pathway SFPs is higher than that of the remaining metabolic SFPs, the pathway shows a bias towards more significant values and vice versa. The tested pathways were “acetyl-CoA biosynthesis“, “aerobic respiration”, “ammonia assimilation cycle“, “ascorbate glutathione cycle”, “flavonoid biosynthesis”, “NAD/NADH phosphorylation and dephosphorylation”, “photosynthesis, light reaction”, “scopoletin biosynthesis”, “simple coumarins biosynthesis”, “sinapate ester biosynthesis”, “tetrahydrofolate biosynthesis” and “tyrosine biosynthesis”.


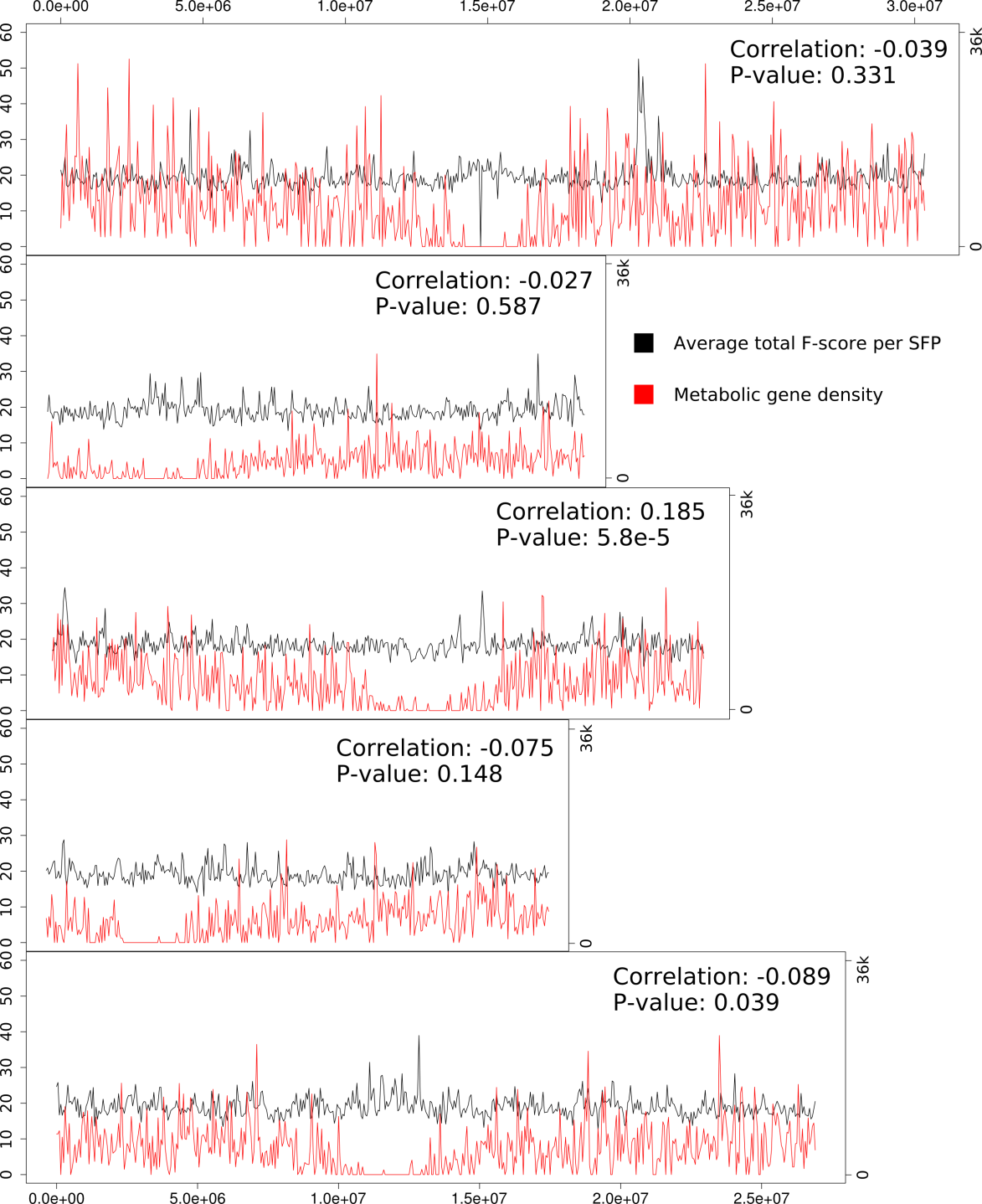


**Figure S4.1:** Correlation of F-score and metabolic gene density. The total F-score across all metabolic trait associations has been calculated for each SFP and averaged in a window of 50,000 nucleotides. The left axis shows the total F-score, the right axis shows the total size of the metabolic genes and the x-axis shows the position in the chromosome. After multiple testing correction, a threshold of 0.01 is considered significant and only chromosome 3 shows a significant positive correlation between F-score and metabolic gene density.

# Genome-wide patterns of genetic variation

## Haplotype structure

B

A


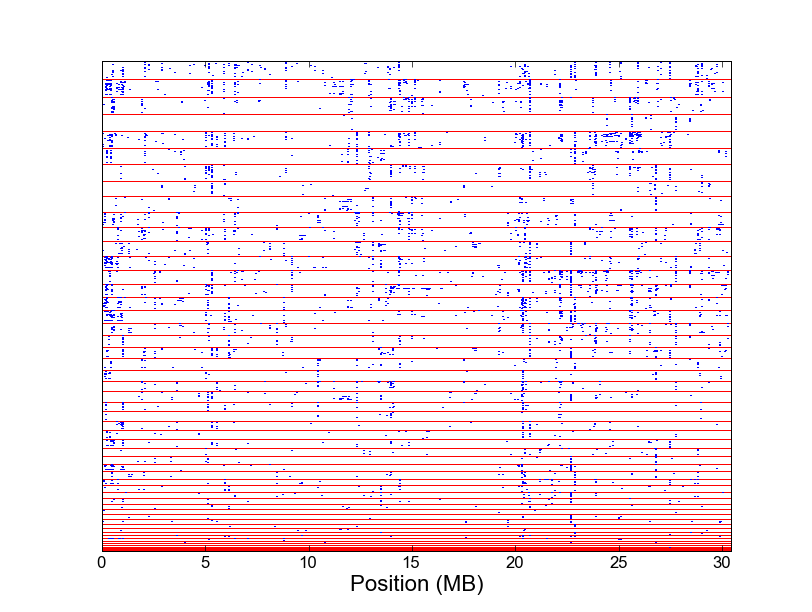


C


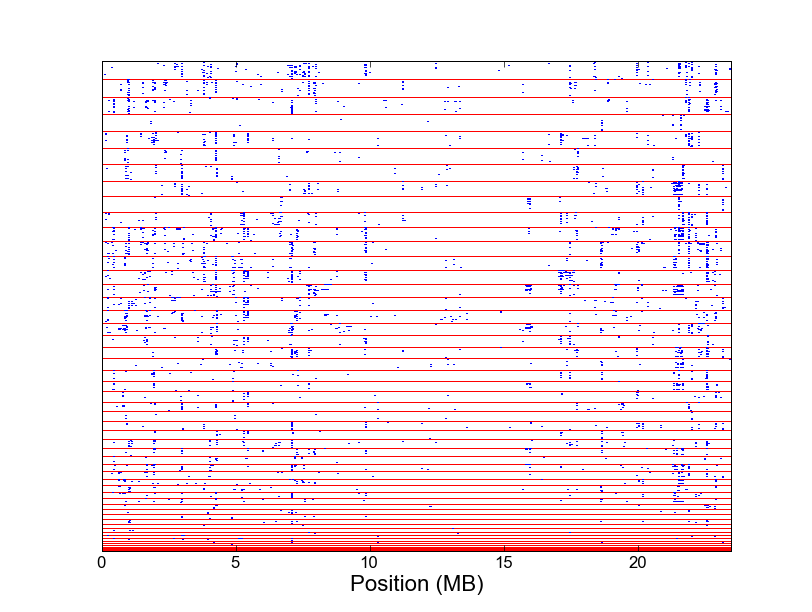


E


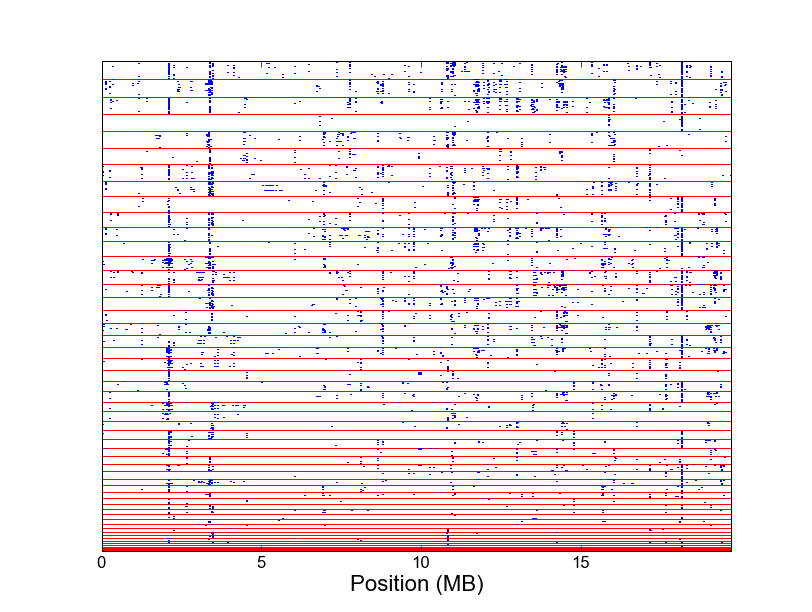


D


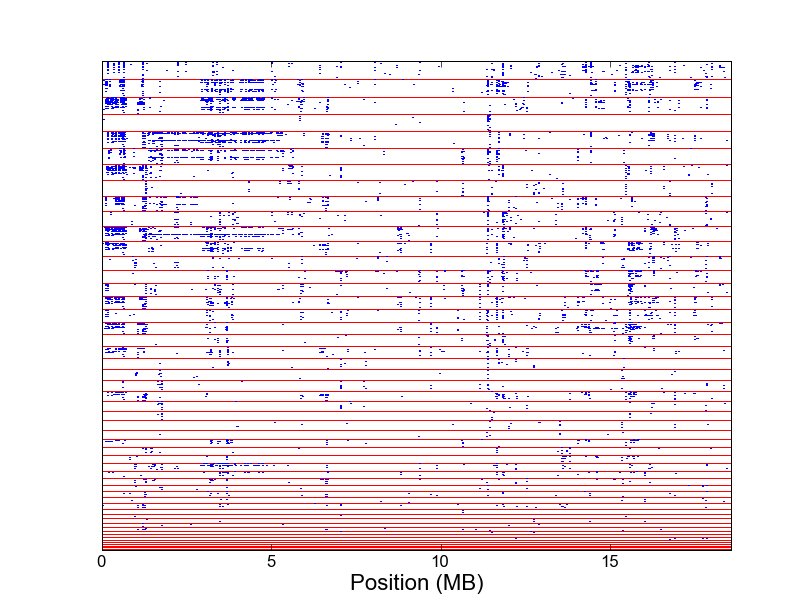


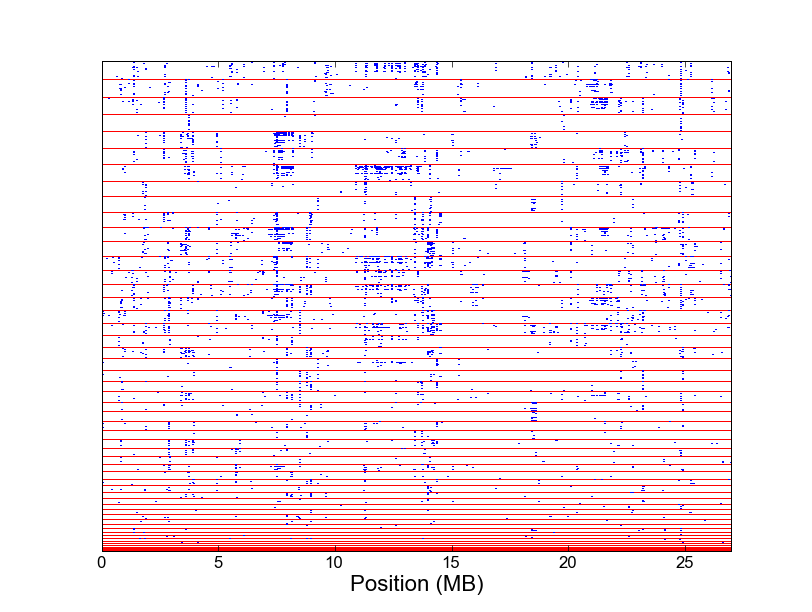


**Figure S5.1A-E:** Pairwise comparison of all accessions. Blue bars show an exact match between two compared 100 SFP windows and thus a shared haplotype in this window. Red lines denote the comparison to the next accession; each possible pair is only shown once. The accessions are plotted in order of their pairwise identity to Col-0, which is the inverted order of the number of called SFPs (see Supplementary Figure 2.2).


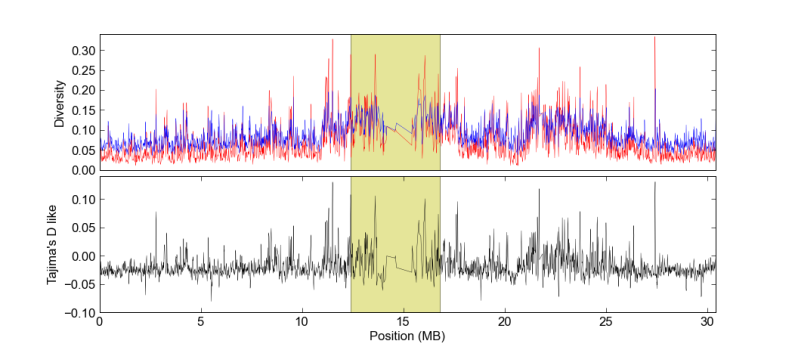


A

B


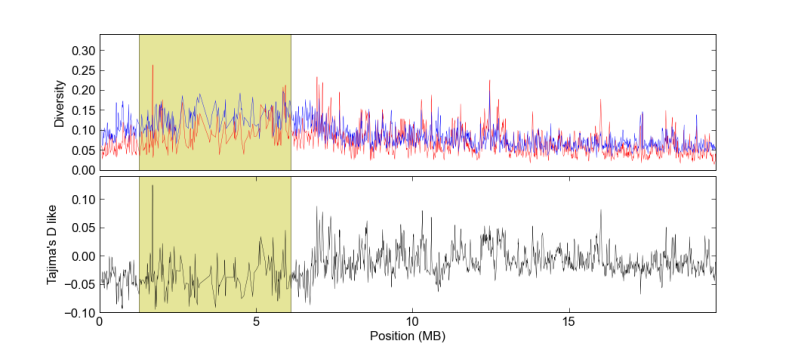


C


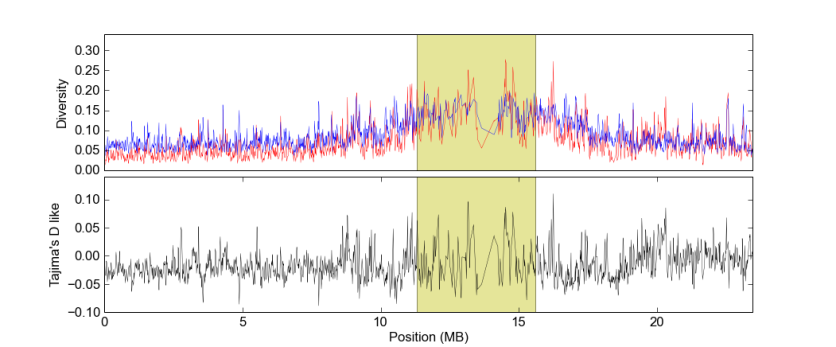


D

**
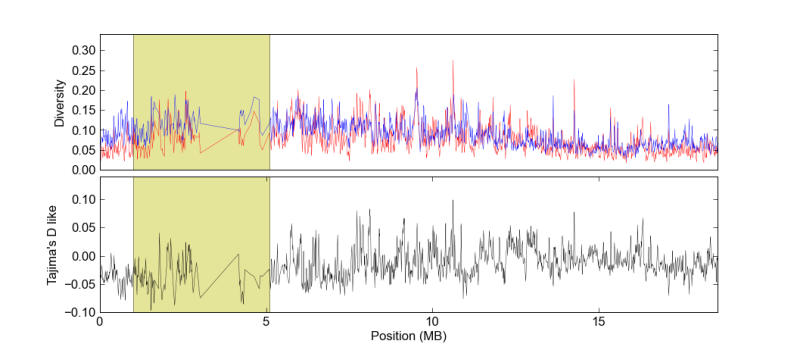
**

E


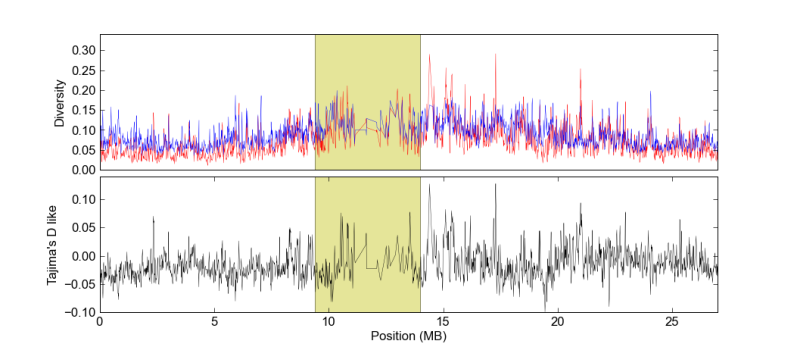


**Figure S5.2A-E:** Nucleotide diversity and frequency spectrum (Tajima's D) for all five chromosomes. The upper graphs show the two diversity measurements π (average pairwise diversity; red) and θ (total diversity; blue) computed in a ±10kb window (containing on average 435 SFPs) around the central base of each gene. The lower graph plots the Tajima's *D*-like statistic [13], which is Tajima's *D* without normalization: π - θ. The green areas indicate the centromeric regions.

A
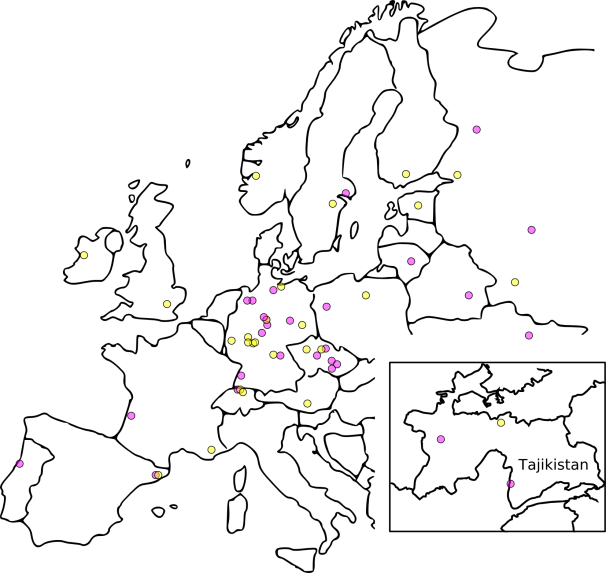
B
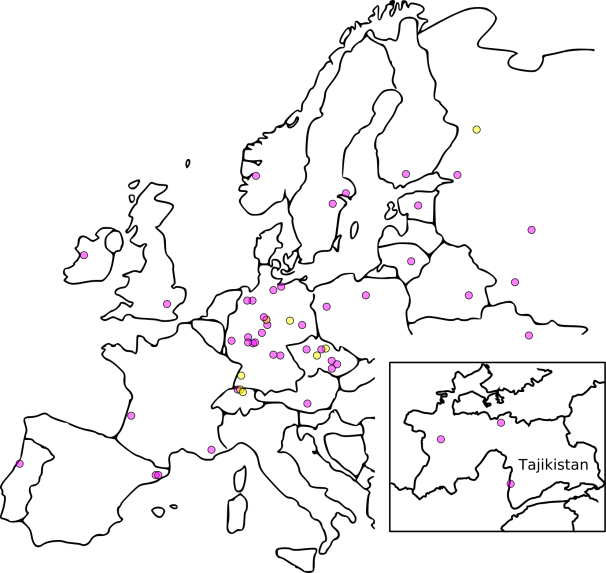


C
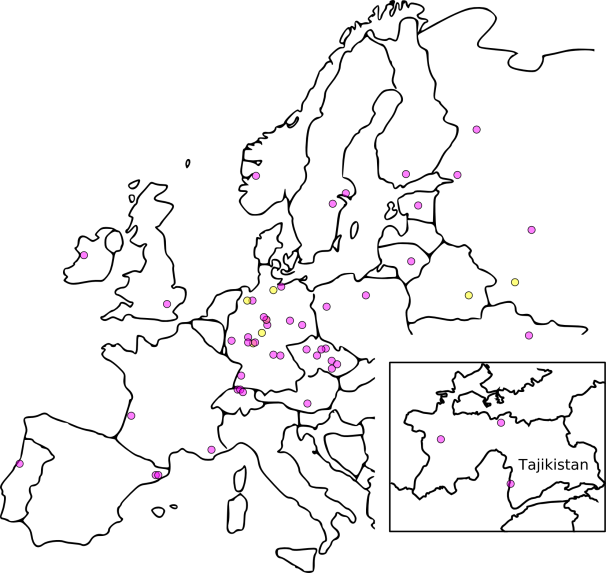
D
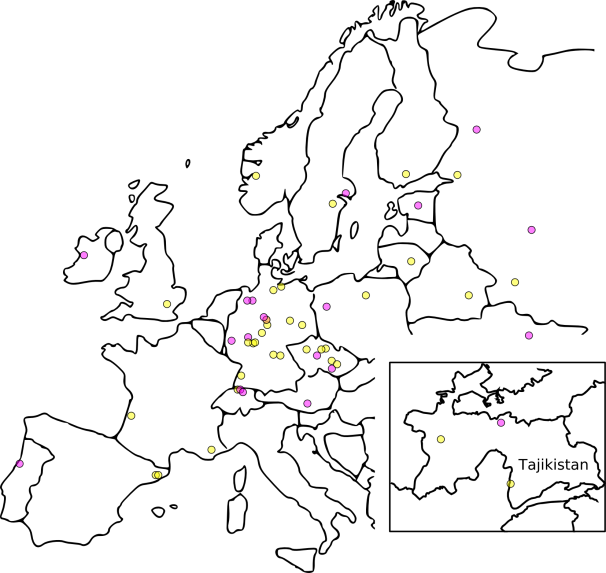


E
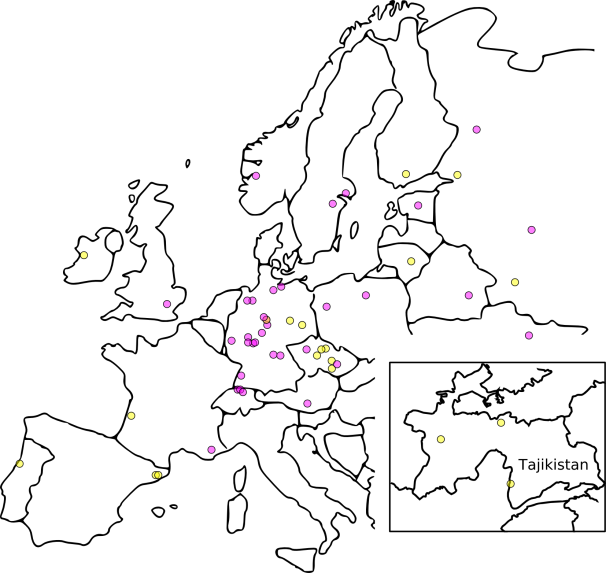


**Figure S5.3A-E:** Sweep locations based on focal SFPs. A-E show the locations of the Col-0 (purple) and non-Col-0 (yellow) genotypes for each sweep SR1-5 respectively. Based on the PHS-test, alleles that are surrounded by the long haplotype are likely to belong to the selected sweep, thus the SR1 is non-Col-0, SR2 – Col-0, SR3 – Col-0, SR4 non-Col-0 and SR5 – non-Col-0. A Mann-Whitney U test comparing the distances of like alleles (determined by the focal SFP) in each sweep against those based on 10,000 shuffled variations, shows that like alleles in sweeps SR2, 3 and 5 have significant neighbouring tendencies with p-values of 3.73e-4, 8.71e-3 and 3.31e-8 respectively. In each case, purple is the Col-0 allele and yellow is the non-Col-0 allele.

## Sweep annotation


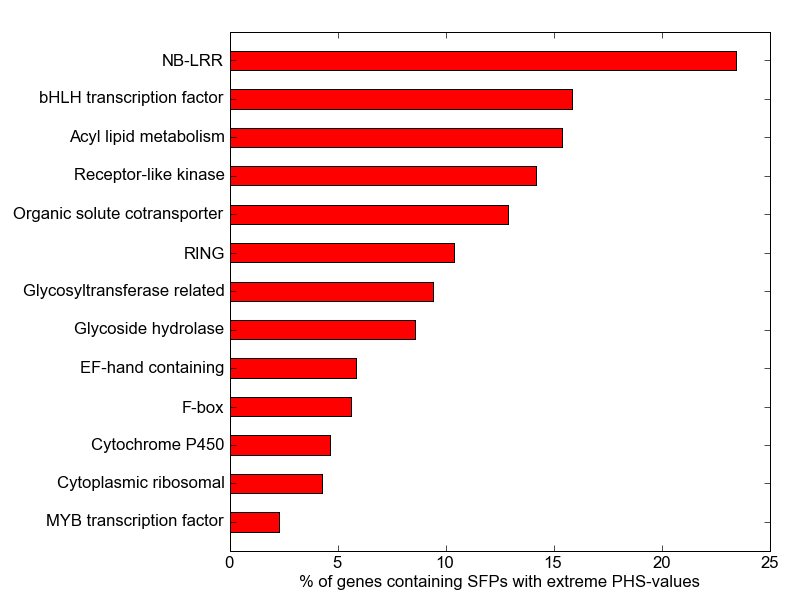


**Figure S5.4:** Relative number of genes belonging to 13 functional classes that contain SFPs with an extreme PHS-value, which shows an unusual extreme ratio between the two haplotypes at this position.


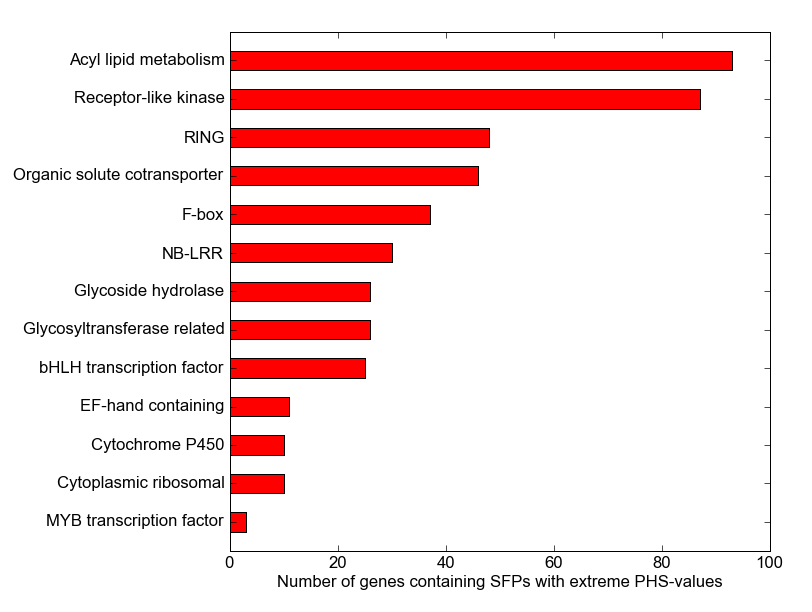


**Figure S5.5:** Absolute number of genes belonging to 13 functional classes that contain an SFPs with an extreme PHS-value, which shows an unusual extreme ratio between the two haplotypes at this position.

| **Pathway** | **p-value** |
| --- | --- |
| **60s ribosomal** | 1 |
| **ABC transporter** | 1 |
| **AP2-EREBP transcription factor** | 1 |
| **ATP binding** | 1 |
| **Acyl lipid metabolism** | 1 |
| **Anthranilate synthase** | 1 |
| **C2H2 transcription factor** | 1 |
| **Calcium dependent protein kinase** | 1 |
| **Calmodulin binding** | 1 |
| **Carbohydrate esterase** | 1 |
| **Chloroplast and mitochondria gene families** | 1 |
| **Cytochrome p450** | 1 |
| **Cytoplasmic ribosomal** | 1 |
| **DC1 domain-containing** | 1 |
| **Disease resistance (NBS-LRR class)** | 0.6865 |
| **EF-hand containing** | 1 |
| **Eukaryotic translation initiation factor** | 1 |
| **F-box** | 1 |
| **Glycoside hydrolase** | 1 |
| **Glycosyl transferase** | 1 |
| **Helicase** | 1 |
| **Kelch repeat-containing** | 1 |
| **Leucine-rich repeat kinase** | 0.4427 |
| **MADS-box transcription factor** | 1 |
| **MYB transcription factor** | 1 |
| **Monooxygenase** | 1 |
| **Organic solute cotransporter** | 1 |
| **Plant cell wall biosynthesis** | 1 |
| **RING** | 1 |
| **Receptor-like kinase** | 0.1671 |
| **SNARE and associated proteins** | 1 |
| **Subtilisin-like serine protease** | 1 |
| **Terpene synthesis** | 1 |
| **Thioredoxin** | 1 |
| **Transmembrane receptor** | 1 |
| **Ubiquitin** | 1 |
| **WRKY transcription factor** | 1 |
| **Wall-associated protein** | 1 |
| **Zinc knuckle (CCHC-type)** | 1 |
| **bHLH transcription factor** | 1 |

**Table S5.1**: Gene families in sweep regions. After multiple testing correction (Benjamini-Hochberg), no significant over-represented gene families can be found in the sweep regions.

**References**

1. Toerjek O, Berger D, Meyer RC, Muessig C, Schmid KJ, Soerensen TR, Weisshaar B, Mitchell-Olds T, Altmann T: **Establishment of a high-efficiency SNP-based framework marker set for Arabidopsis**. *Plant Journal* 2003, **36**(1):122-140.

2. Gibon Y, Blaesing OE, Hannemann J, Carillo P, Hohne M, Hendriks JHM, Palacios N, Cross J, Selbig J, Stitt M: **A robot-based platform to measure multiple enzyme activities in Arabidopsis using a set of cycling assays: Comparison of changes of enzyme activities and transcript levels during diurnal cycles and in prolonged darkness**. *Plant Cell* 2004, **16**(12):3304-3325.

3. Cross JM, von Korff M, Altmann T, Bartzetko L, Sulpice R, Gibon Y, Palacios N, Stitt M: **Variation of enzyme activities and metabolite levels in 24 Arabidopsis accessions growing in carbon-limited conditions**. *Plant Physiology* 2006, **142**(4):1574-1588.

4. Schauer N, Semel Y, Roessner U, Gur A, Balbo I, Carrari F, Pleban T, Perez-Melis A, Bruedigam C, Kopka J *et al*: **Comprehensive metabolic profiling and phenotyping of interspecific introgression lines for tomato improvement**. *Nature Biotechnology* 2006, **24**(4):447-454.

5. Lisec J, Schauer N, Kopka J, Willmitzer L, Fernie AR: **Gas chromatography mass spectrometry-based metabolite profiling in plants**. *Nature Protocols* 2006, **1**(1):387-396.

6. Schauer N, Steinhauser D, Strelkov S, Schomburg D, Allison G, Moritz T, Lundgren K, Roessner-Tunali U, Forbes MG, Willmitzer L *et al*: **GC-MS libraries for the rapid identification of metabolites in complex biological samples**. *Febs Letters* 2005, **579**(6):1332-1337.

7. Warthmann N, Fitz J, Weigel D: **MSQT for choosing SNP assays from multiple DNA alignments**. *Bioinformatics* 2007, **23**(20):2784-2787.

8. Hubisz MJ, Falush D, Stephens M, Pritchard JK: **Inferring weak population structure with the assistance of sample group information**. *Molecular Ecology Resources* 2009, **9**(5):1322-1332.

9. Nordborg M, Hu TT, Ishino Y, Jhaveri J, Toomajian C, Zheng H, Bakker E, Calabrese P, Gladstone J, Goyal R *et al*: **The pattern of polymorphism in *Arabidopsis thaliana***. *PLoS Biology* 2005, **3**(7):e196.

10. Hofacker IL, Fontana W, Stadler PF, Bonhoeffer SL, Tacker M, Schuster P: **Fast Folding and Comparison of RNA Secondary Structures**. *Monatsh Chem* 1994, **125**:167--188.

11. Tusher VG, Tibshirani R, Chu G: **Significance analysis of microarrays applied to the ionizing radiation response**. *Proc Natl Acad Sci U S A* 2001, **98**(9):5116-5121.

12. Clark RM, Schweikert G, Toomajian C, Ossowski S, Zeller G, Shinn P, Warthmann N, Hu TT, Fu G, Hinds DA *et al*: **Common sequence polymorphisms shaping genetic diversity in *Arabidopsis thaliana***. *Science* 2007, **317**(5836):338-342.

13. Borevitz JO, Hazen SP, Michael TP, Morris GP, Baxter IR, Hu TT, Chen H, Werner JD, Nordborg M, Salt DE *et al*: **Genome-wide patterns of single-feature polymorphism in *Arabidopsis thaliana***. *Proc Natl Acad Sci U S A* 2007, **104**(29):12057-12062.
